# Bioelectrosynthesis of Signaling Molecules for Selective Modulation of Cell Signaling

**DOI:** 10.1002/anie.202508192

**Published:** 2025-08-04

**Authors:** Myeongeun Lee, Jaewoong Lee, Yongha Kim, Changho Lee, Sang Yeon Oh, Jihan Kim, Jimin Park

**Affiliations:** ^1^ Department of Chemical and Biomolecular Engineering Korea Advanced Institute of Science and Technology (KAIST) Daejeon 34141 South Korea

**Keywords:** Biocatalysis, Bioelectrosynthesis, Computational chemistry, Electrochemistry, Signal transduction

## Abstract

Bioelectrosynthesis holds great potential for studying and regulating biological systems through the in situ synthesis and delivery of cell signaling molecules with high spatiotemporal precision. Despite recent advancements, precise control over multiple signaling molecules within a single platform remains challenging. Here, we introduce a bioelectrosynthesis approach capable of selectively producing two types of signaling molecules from a single precursor. This system leverages multi‐metal sulfide electrocatalysts inspired by denitrifying enzymes, which generate signaling molecules, nitric oxide (NO), and ammonia (NH_3_) from nitrite ions. By controlling catalytic active sites, NO or NH_3_ can be selectively produced under mild electric fields in physiologically relevant conditions. In situ product analyses and first‐principles calculations reveal that NO intermediate binding affinity determines product selectivity. These electrocatalysts integrate seamlessly with biological systems, allowing precise, on‐demand modulation of NO‐ or NH_3_‐mediated signaling pathways in human cell lines. By combining electrochemical precision with selective cell control, this strategy may advance the study and regulation of biological systems.

## Graphical abstract

A bioelectrosynthesis platform is devised for electrochemical control of cellular signaling under physiological conditions. The system allows on‐demand, selective generation of signaling molecules, nitric oxide (NO) and ammonia (NH_3_), from nitrite (NO_2_^−^) ions using Cu_2_MoS_4_‐based electrocatalysts. This approach enables independent, electrochemical modulation of NO‐ or NH_3_‐sensitive ion channels expressed in genetically engineered cells.

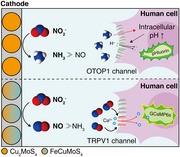

## Introduction

Bioelectrosynthesis of signaling molecules has gained significant attention in biomedical research, including cell biology, tissue engineering, and cancer therapy.^[^ ^1^, ^2^, ^3^ ^]^ By leveraging the high spatiotemporal resolution and quantitative nature of electrochemistry, bioelectrosynthesis allows for the precise and targeted delivery of these signaling molecules to the targeted cells.^[^ ^4^ ^]^ In particular, this system offers a distinct advantage in controlling chemically unstable or gaseous signaling molecules, which are challenging to regulate with traditional methods, such as direct injection or the use of materials that release these molecules.^[^ ^5^, ^6^ ^]^

Prior research demonstrated the feasibility of bioelectrosynthesis for the controlled generation of a diverse range of transient or gaseous signaling molecules, including nitric oxide (NO), carbon monoxide (CO), and reactive oxygen species (ROS), in biological solutions.^[^ ^2^, ^3^, ^4^, ^7^, ^8^ ^]^ These approaches enabled the direct modulation of signaling pathways mediated by these molecules in various cell types and animal organs. These results highlight the potential of bioelectrosynthesis as a powerful tool for studying dynamic cellular processes mediated by these transient molecules.

Current bioelectrosynthesis approaches, however, often rely on different precursors to generate distinct signaling molecules.^[^ ^7^, ^9^ ^]^ Exchanging precursors inside biological systems poses several challenges, including residual precursor interference, disruption of cellular environments during the exchange, and low temporal resolution. Consequently, current methods face significant limitations in effectively regulating multiple signaling molecules within a single system. Given that biological signaling pathways are intricate networks mediated by multiple signaling molecules, the development of an advanced bioelectrosynthesis approach capable of synthesizing multiple signaling molecules without changing precursors is necessary.

Here we propose a concept of multiplexed bioelectrosynthesis, where multiple signaling molecules can be selectively synthesized from the single precursor, offering independent and simultaneous control of signaling pathways. To demonstrate a proof‐of‐concept of our bioelectrosynthesis approach, we leveraged biological denitrification processes, in which the nitrite anion (NO_2_^−^) can be reduced to multiple signaling molecules, including NO, hydroxylamine (NH_2_OH), and ammonia (NH_3_).^[^ ^10^, ^11^, ^12^, ^13^ ^]^ Among these, NO and NH_3_ are co‐involved in various physiological processes, including neurotransmission and vascular regulations.^[^ ^14^, ^15^, ^16^, ^17^ ^]^ For instance, NH_3_ is integral to the glutamate–glutamine cycle in astrocytes, a process closely linked to NO production in neighboring neurons.^[^ ^18^, ^19^, ^20^, ^21^ ^]^ Additionally, both NO and NH_3_ influence the levels of cyclic guanosine monophosphate (cGMP), a secondary messenger involved in vascular homeostasis and neurotransmission.^[^ ^18^, ^22^, ^23^, ^24^, ^25^ ^]^ Given the intricate interplay between these molecules, achieving precise and localized control over their synthesis in a single platform is essential for studying and manipulating complex biological networks.

By taking inspiration from these biological NO_2_^−^ reduction reactions, we aim to develop a bioelectrosynthesis approach allowing for the in situ generation of two signaling molecules, NO and NH_3_, from a single NO_2_^−^ precursor (Equations 1 and 2).

(1)

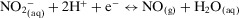



(2)

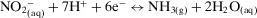




**Figure 1:**
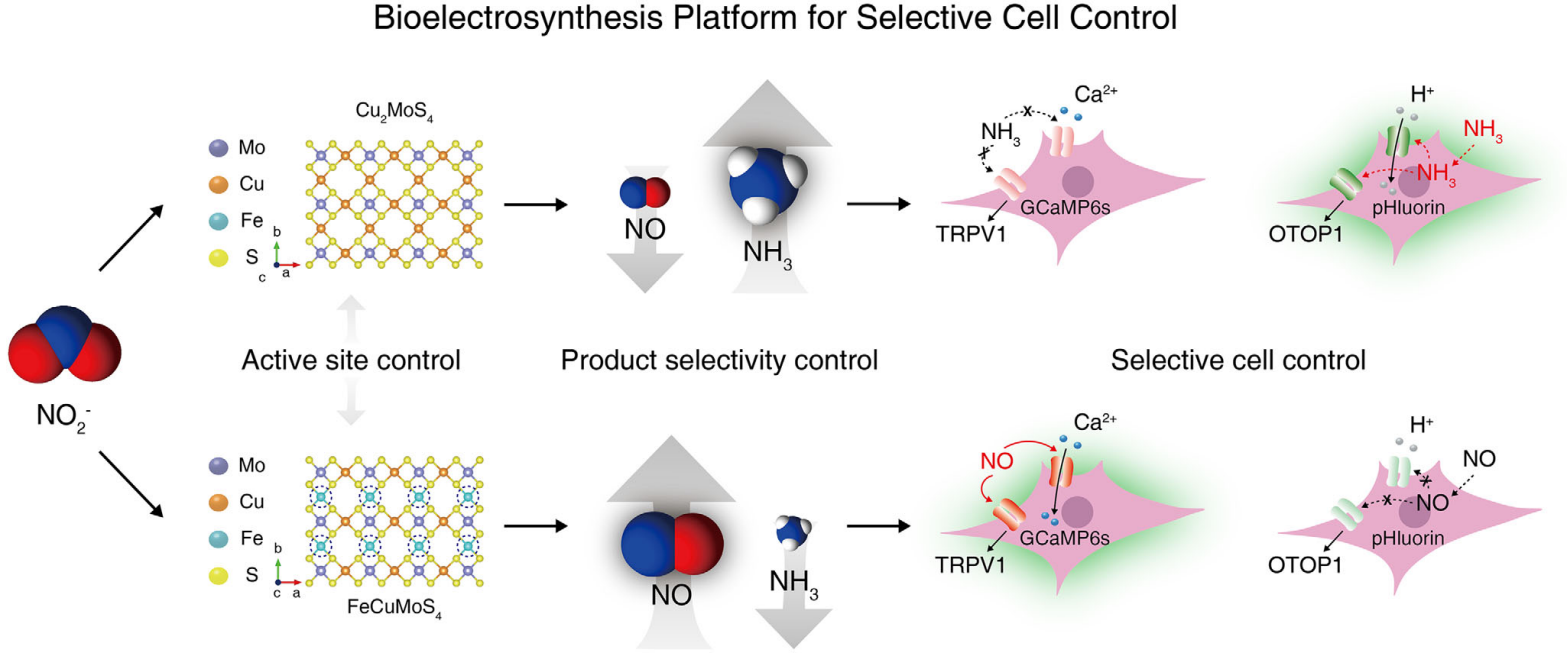
A schematic of a bioelectrosynthesis approach for the independent and selective production of signaling molecules from the NO_2_^−^ ion. The control of active sites in the electrocatalysts determines the selectivity toward NO and NH_3_, offering an independent modulation of corresponding cell signaling.

We employed Cu_2_MoS_4_, a 2D ternary chalcogenide crystal, as a model electrocatalyst for NO_2_^−^ reduction reaction based on its structural and compositional similarities to metalloenzymes involved in the denitrification reaction.^[^ ^13^, ^26^, ^27^, ^28^ ^]^ Biological metalloenzymes include Fe‐ or Cu‐based nitrite reductase, Mo‐based xanthine oxidase, and Fe–Mo nitrogenase, where transition metal centers (Fe, Mo, and Cu) are ligated by the sulfur atoms.^[^ ^13^, ^29^, ^30^, ^31^, ^32^ ^]^ The structural flexibility of Cu_2_MoS_4_ crystal enabled the facile doping of other transition metal ions, including Fe ions.^[^ ^33^, ^34^, ^35^ ^]^ It was found that these Cu_2_MoS_4_‐based electrocatalysts can convert NO_2_^−^ ion precursors into either NO or NH_3_ signaling molecules, and product selectivity can be greatly changed by the Fe doping process. These electrocatalysts were implemented into an electrosynthesis platform and enabled selective activation of NO‐ or NH_3_‐sensitive ion channels in vitro (Figure 1).

## Results and Discussion

### Development of Cu_2_MoS_4_‐Based Catalysts for Bioelectrosynthesis of NO and NH_3_

**Figure 2:**
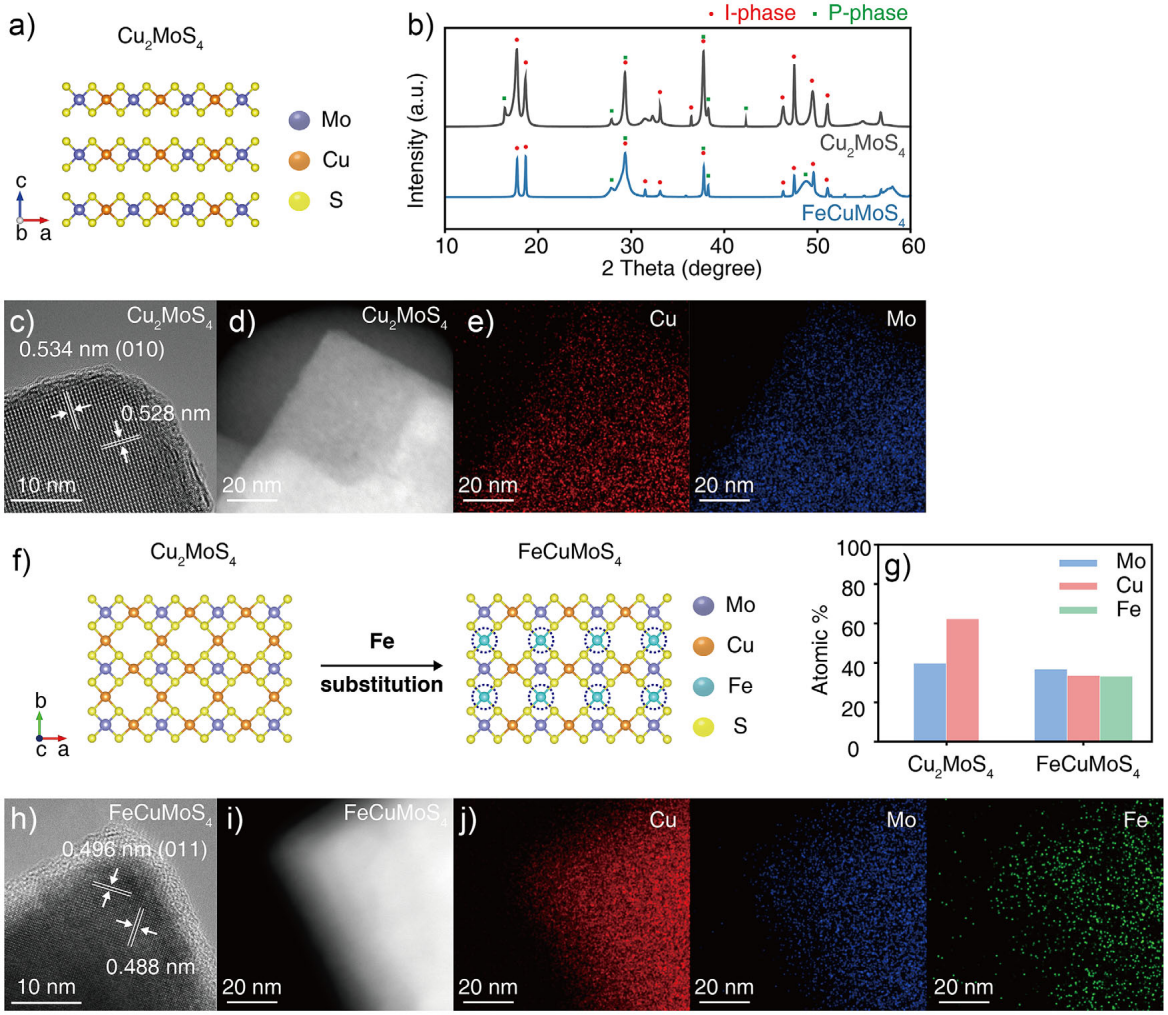
a) Schematic representation of the crystal structure of Cu_2_MoS_4_. Mo, Cu, and S atoms are marked in purple, orange, and yellow, respectively. b) XRD patterns for synthesized Cu_2_MoS_4_ and FeCuMoS_4_. c) HRTEM, d) STEM, and e) STEM‐EDS mapping images of synthesized Cu_2_MoS_4_. f) A schematic of the Fe doping process for Cu_2_MoS_4_ to synthesize FeCuMoS_4_. Doped Fe ions are depicted in dotted circles. g) ICP‐OES results of Cu_2_MoS_4_ and FeCuMoS_4_ indicated that doped Fe ions preferentially substituted the Cu ions. h) HRTEM, i) STEM, and j) STEM‐EDS mapping images of FeCuMoS_4_. The STEM‐EDS mapping image indicated uniform distributions of Cu (red), Mo (blue), and Fe (green) ions inside the crystal.

The Cu_2_MoS_4_ crystals were synthesized using a solvothermal method.^[^ ^36^ ^]^ Similar to the metalloenzymes found in biological denitrification reactions, the Cu_2_MoS_4_ crystals have layered structures where Cu and Mo atoms are alternatively connected by sulfur anions (Figure 2a). Powder X‐ray diffraction (XRD) analysis demonstrated that the synthesized Cu_2_MoS_4_ crystals predominantly have an I‐phase with the formation of some minor P‐phase (Figure 2b). Scanning electron microscopy (SEM) and high‐resolution transmission electron microscopy (HRTEM) analyses also confirmed that the Cu_2_MoS_4_ crystals are predominantly comprised of the I‐phase, as seen in the layered morphology of the crystals (Figures 2c and S1).^[^ ^37^ ^]^ HRTEM analyses further showed that the crystal has an interplanar spacing of 0.534 and 0.528 nm, which refers to the (010) crystal plane of the I‐phase. Scanning transmission electron microscopy equipped with energy‐dispersive X‐ray spectroscopy (STEM‐EDS) analysis showed that Cu and Mo atoms are uniformly distributed inside the crystal (Figure 2c–e).

Due to the structural flexibility and presence of multivalent metal ions (Cu^+^/Cu^2+^ and Mo^4+^/Mo^6+^), Cu_2_MoS_4_ crystals can be readily doped with another type of transition metal ions, Fe ions, which have been shown to be involved in the biological denitrification process (Figure 2f).^[^ ^28^, ^38^ ^]^ XRD results showed that these Cu_2_MoS_4_ crystals can retain their crystal structure until 33 at% Fe ions are doped inside the crystal (Figure 2b). Further addition of the Fe ions resulted in the formation of the secondary phase, CuFe_2_S_3_ (Figure S2). Inductively coupled plasma optical emission spectroscopy (ICP‐OES) analysis showed that doped Fe ions preferentially substituted Cu ions inside the crystal. Doping 33 at% Fe ions inside Cu_2_MoS_4_ resulted in the formation of the FeCuMoS_4_ crystals with the equimolar distribution of Cu, Fe, and Mo ions (Figure 2g). SEM and HRTEM images of FeCuMoS_4_ crystals showed that these crystals have layered morphologies similar to those of Cu_2_MoS_4_ crystals (Figures 2h,i and S1). STEM‐EDS mapping further confirmed the uniform distribution of Fe ions throughout the FeCuMoS_4_ crystal (Figure 2j).

### Selective Electrosynthesis of NO and NH_3_ in Biological Solutions

**Figure 3:**
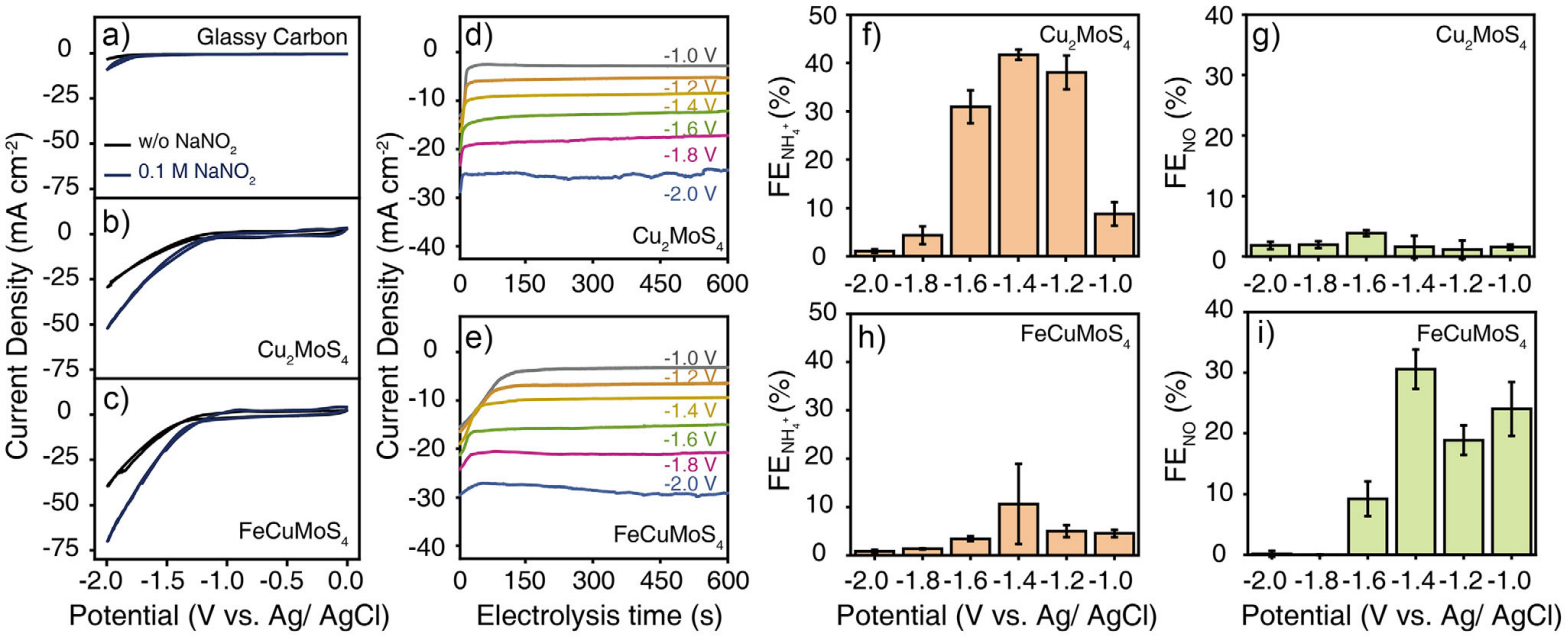
CV curves of the a) bare carbon, b) Cu_2_MoS_4_‐loaded, and c) FeCuMoS_4_‐loaded electrodes in the presence or absence of 0.1 M NO_2_^−^ containing Tyrode's solution at a scan rate of 50 mV s^−1^. Chronoamperometry profiles of d) Cu_2_MoS_4_‐loaded and e) FeCuMoS_4_‐loaded electrodes at the applied voltage from −1.0 to −2.0 V versus Ag/AgCl. FE_NH4+_ (mean ± SD) with the f) Cu_2_MoS_4_ or h) FeCuMoS_4_ electrocatalysts and FE_NO_ (mean ± SD) with the g) Cu_2_MoS_4_ or i) FeCuMoS_4_ electrocatalysts at various applied voltage conditions, respectively (n = 3 independent experiments per group). A potential range from −1.0 to −2.0 V versus Ag/AgCl was utilized for the analysis.

We then assessed the electrocatalytic activities of Cu_2_MoS_4_ and FeCuMoS_4_ for the NO_2_^−^ reduction reaction. The electrocatalytic performance was assessed in a three‐compartment electrochemical cell containing Tyrode's solution, a simulated physiological solution, with 0.1 M NaNO_2_ at pH 7.4. Here, cathodes were fabricated by loading Cu_2_MoS_4_ or FeCuMoS_4_ crystals onto carbon electrodes, whereas platinum (Pt) and Ag/AgCl electrodes served as the anode and reference electrodes, respectively. Cyclic voltammetry (CV) curves of Cu_2_MoS_4_‐ or FeCuMoS_4_‐loaded carbon electrodes exhibited significantly higher Faradaic currents in NaNO_2_‐containing Tyrode's solution compared to the bare carbon electrodes (Figure 3a–c). These Faradaic currents increased as the concentration of NO_2_^−^ inside the solution increased, indicating that NO_2_^−^ is the predominant reactant during the electrocatalysis (Figure S3).

To identify the reaction products generated from the Cu_2_MoS_4_‐ or FeCuMoS_4_‐loaded carbon electrodes, we performed chronoamperometry analysis at the wide range of applied voltage from −1.0 to −2.0 V versus Ag/AgCl (Figure 3d,e). After the chronoamperometry analysis, the electrolyte was collected, followed by the quantification of generated NO, NH_3_, and NH_2_OH. For NO quantification, the NO‐sensitive fluorescent probe, 4‐amino‐5‐methylamino‐2′,7′‐difluorofluorescein (DAF‐FM), which can form a fluorescent benzotriazole derivative upon reaction with NO, was employed. In the case of NH_3_, the product amount was quantified using the salicylate method, whereas the amount of NH_2_OH was measured with the spectrophotometric method.^[^ ^39^, ^40^, ^41^ ^]^

Interestingly, Cu_2_MoS_4_ and FeCuMoS_4_ electrocatalysts showed significantly different product selectivity for NO_2_^−^ reduction reaction despite their comparable Faradaic current levels. Cu_2_MoS_4_ electrocatalysts predominantly converted NO_2_^−^ into NH_3_ at the potential range from −1.2 to −1.6 V versus Ag/AgCl, while a negligible amount of NO was detected at the same applied voltages (Figure 3f,g). In contrast, the production of NH_3_ was largely suppressed in the case of FeCuMoS_4_ electrocatalysts. FeCuMoS_4_ electrocatalysts, instead, produced significantly higher amounts of NO compared to the Cu_2_MoS_4_ electrocatalysts under the same applied voltages (Figure 3h,i). Here, the Faradaic efficiency (FE) for each reaction product was calculated by relating the total charge passed during chronoamperometry to the amount of products captured after the chronoamperometry, considering the number of electrons involved in each reaction (Equations 1 and 2). Note that the measured FE toward NO and NH_3_ here is a lower‐bound value considering their gaseous nature. Moreover, we expect that the autoxidation process of NO resulted in the further underestimation of FE for NO, especially in the case of FeCuMoS_4_ electrocatalysts, considering that the autoxidation kinetics is proportional to the square of the NO concentration.^[^ ^42^, ^43^ ^]^

The FE toward NH_2_OH (FE_NH2OH_) was below 1% at the applied voltage range of −1.0 to −2.0 V versus Ag/AgCl in both electrocatalysts, indicating the negligible production of NH_2_OH byproduct (Figure S4). At significantly negative applied potentials (≤−1.8 V), both FE_NO_ and FE_NH4+_ decreased, implying that the hydrogen evolution reaction (HER) became the predominant reaction over the NO_2_^−^ reduction reaction. Put together, we verified that Cu_2_MoS_4_ and FeCuMoS_4_ electrocatalysts can be utilized to selectively convert NO_2_^−^ precursors into NH_3_ and NO signaling molecules, respectively.

### Mechanistic Studies on Bioelectrosynthesis of NO and NH_3_

**Figure 4:**
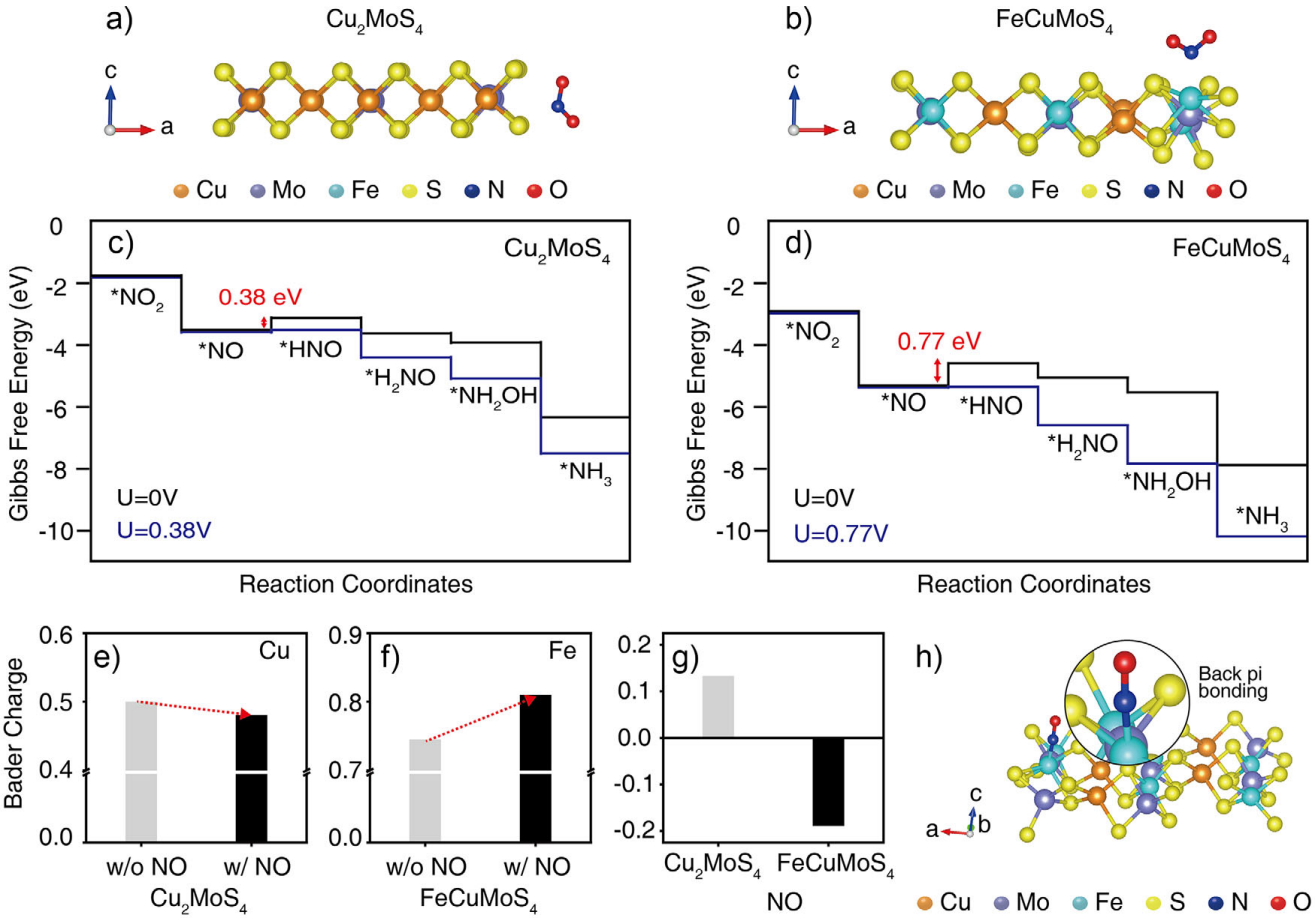
Configurations illustrating the binding of NO_2_^−^ to a) Cu_2_MoS_4_ and b) FeCuMoS_4_ surfaces. Cu, Mo, Fe, S, N, and O atoms were marked in orange, purple, cyan, yellow, blue, and red circles, respectively. c) Gibbs free energy diagrams for the NO_2_^−^ reduction reaction catalyzed by Cu_2_MoS_4_ at an applied potential (U) of 0 V (black) and 0.38 V versus the RHE (blue). d) Gibbs free energy diagrams for the NO_2_^−^ reduction reaction catalyzed by FeCuMoS_4_ at U = 0 V (black) and 0.77 V versus RHE (blue). Atomic Bader charge analyses for e) Cu in Cu_2_MoS_4_ and f) Fe in FeCuMoS_4_ before (gray) and after (black) binding to NO. g) Changes in Bader charge of NO after the binding to Cu_2_MoS_4_ and FeCuMoS_4_. h) A schematic illustrating the strong back‐pi bonding between the Fe active site and NO intermediates in FeCuMoS_4_.

To understand the mechanism responsible for the distinct product selectivity observed between Cu_2_MoS_4_ and FeCuMoS_4_, we performed density functional theory (DFT) simulations. Based on our XRD and TEM results, we used a well‐defined I‐phase structure of Cu_2_MoS_4_ in our simulation. To model the structure of FeCuMoS_4_, we performed a series of calculations where Fe ions are doped into different Cu sites of Cu_2_MoS_4_. Among the diverse possible structures of FeCuMoS_4_, we chose the most thermodynamically stable structure for our simulation (Figures 2f and S5 and Table S1). In these structures, the edge plane configuration had the most stable binding energy between the NO_2_^−^ ions and electrocatalysts, implying that NO_2_^−^ reduction presumably occurs at the edge sites (Figures 4a,b and S6 and Table S2).

After defining the structure of Cu_2_MoS_4_ and FeCuMoS_4_, we calculated the Gibbs free energy changes in Cu_2_MoS_4_ and FeCuMoS_4_ edge sites during the NO_2_^−^ reduction. Gibbs free energy calculations revealed a reaction barrier associated with the conversion of NO to HNO at 0 V versus the reversible hydrogen electrode (RHE), similar to the reaction mechanism found in other NO_2_^−^ reduction electrocatalysts.^[^ ^44^ ^]^ The voltage required to overcome this barrier was calculated to be 0.38 V for Cu_2_MoS_4_ electrocatalyst, whereas a significantly higher voltage (0.77 V) was theoretically necessary to overcome the same reaction barrier for FeCuMoS_4_ (Figure 4c,d). These findings implied that the subsequent reduction of NO toward NH_3_ can be relatively facilitated in Cu_2_MoS_4_ compared to FeCuMoS_4_ at the same applied voltages. Indeed, our experimental results showed that Cu_2_MoS_4_ produces a relatively larger amount of NH_3_ compared to FeCuMoS_4_ at the same applied voltages (Figure 3f–i).

The absence of additional reaction barriers beyond the NO to HNO transition in our calculations implied that interaction between NO and the metal active sites could play important roles in determining the product selectivity of each electrocatalyst. To understand the interaction between NO intermediates and the metal active sites, we performed Bader charge analyses. In the case of Cu_2_MoS_4_, the binding of NO to Cu resulted in a slight increase in the electron density of Cu (−0.019 of Bader charge) (Figure 4e). In contrast, a significant electron depletion at the Fe site (+0.065 of Bader charge) was found after the binding of NO in the case of FeCuMoS_4_, indicating the strong interaction between Fe ions and NO intermediates in FeCuMoS_4_ (Figure 4f). Accordingly, the Bader charge of NO slightly increased upon binding to the Cu site in the Cu_2_MoS_4_, whereas a robust decrease in the Bader charge of NO was observed after binding to the Fe in the FeCuMoS_4_ (Figure 4g).

Density of states (DOS) analyses corroborated the strong interaction between NO and Fe sites in the FeCuMoS_4_. The d orbitals of the Fe site from FeCuMoS_4_ overlapped with the p orbitals of the N atom from NO near the Fermi level, indicating the presence of strong back‐pi bonding between Fe and NO (Figures 4h and S7). Together, our calculations demonstrated that the doped Fe ions, which can strongly bind to the NO intermediates, play crucial roles in determining the product selectivity for NO_2_^−^ reduction reactions.

### Bioelectrosynthesis of NO and NH_3_ for Selective Cell Control

To demonstrate the feasibility of our system for selective modulation of cell signaling, we first utilized transient receptor potential vanilloid family member 1 (TRPV1) ion channels as model platforms. TRPV1 ion channels are widely expressed in the nervous, epithelial, and gustatory systems, and these ion channels are known to recognize diverse chemical stimuli, including NO, but not NH_3_.^[^ ^45^ ^]^

**Figure 5:**
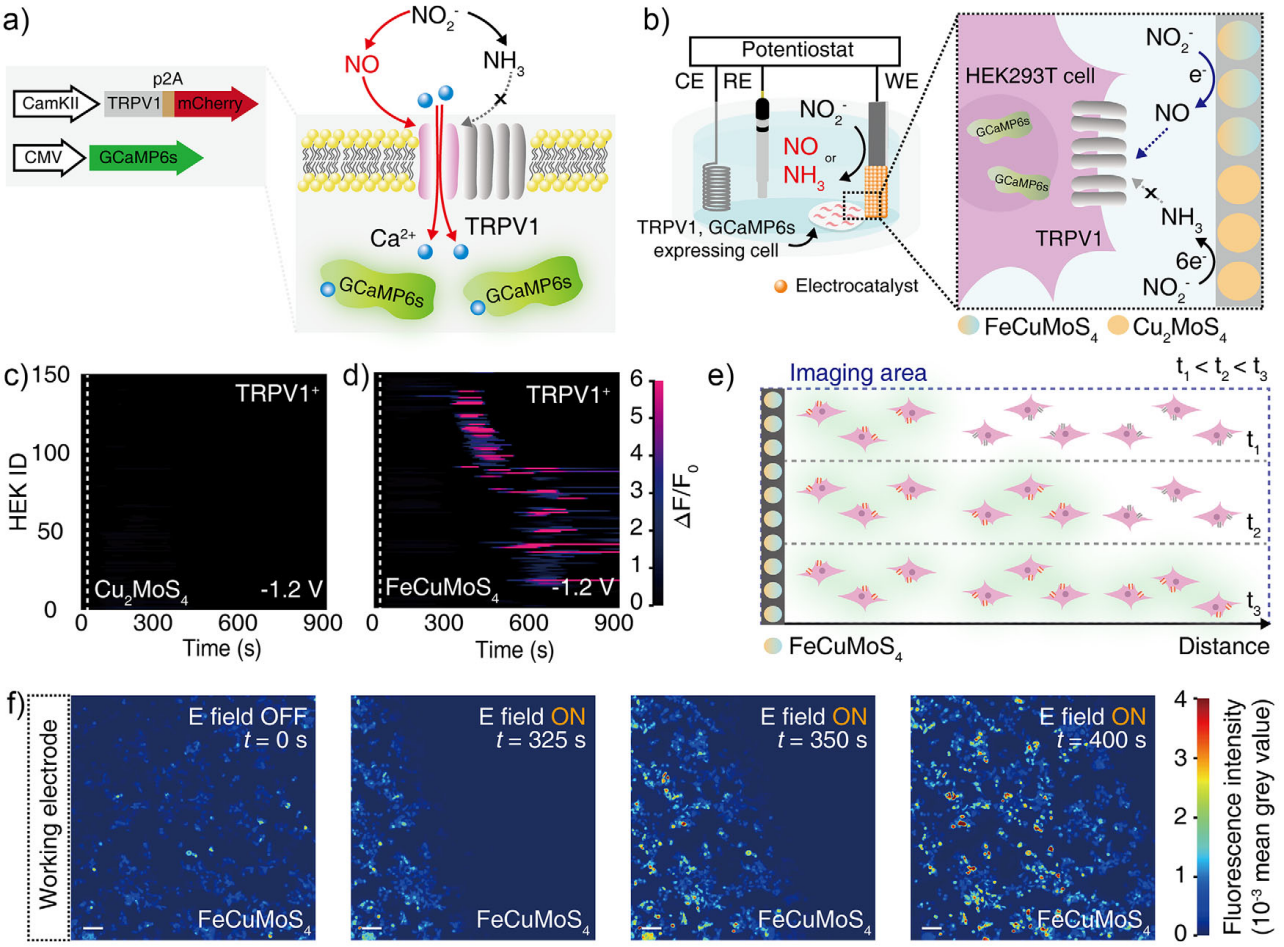
TRPV1 activation with electrochemically produced NO. a) Schematic representation of two plasmids used to generate genetically engineered cells co‐expressing TRPV1 and GCaMP6s (left) and the mechanism of NO‐mediated activation of TRPV1 and subsequent Ca^2+^ influx (right). b) Schematic illustrating selective activation of TRPV1 by electrochemically produced NO, but not by NH_3_. Here, cells were positioned near the electrode where localized electrosynthesis of NO or NH_3_ occurred. Individual GCaMP6s fluorescence traces for 150 TRPV1^+^ cells upon application of a voltage of −1.2 V versus Ag/AgCl to c) Cu_2_MoS_4_ and d) FeCuMoS_4_ electrocatalysts. The voltages were turned on at 30 s (dashed lines), and continuous electrochemical synthesis was performed until 900 s. Here, HEK ID represents individual cells analyzed, and each horizontal line displays the GCaMP6s fluorescence changes of the individual cell over time. A total of 150 cells were analyzed for each condition (*n* = 3 independent experiments per group). e) Schematic depiction of time‐lapse imaging showing sequential activation of TRPV1^+^ cells during electrosynthesis of NO. Time points t_1_, t_2_, and t_3_ represent increasing durations of electrolysis, with TRPV1^+^ cells located farther from the electrode being activated at later time points due to diffusion of NO. f) Representative time‐lapse images of Ca^2+^ responses in TRPV1^+^ cells in the presence of applied voltages of −1.2 V versus Ag/AgCl and FeCuMoS_4_ electrocatalysts. Electrodes loaded with FeCuMoS_4_ electrocatalysts were located at the left in all four images (scale bar: 100 µm). The vertical axis corresponds to GCaMP6s fluorescence intensity, and the color scale was utilized to visualize the GCaMP6s fluorescence changes.

For in vitro demonstration, human embryonic kidney (HEK) 293T cells were co‐transfected with two plasmids: one encoding TRPV1 linked to the fluorescent protein mCherry via a post‐transcriptional cleavage linker p2A under the excitatory neuronal promoter CaMKIIα (CaMKIIα::TRPV1‐p2A‐mCherry), and another encoding the genetically encoded fluorescent calcium ion (Ca^2+^) indicator GCaMP6s under the broad cytomegalovirus promoter (CMV::GCaMP6s). Here, mCherry was served as a marker to confirm TRPV1 expression, whereas GCaMP6s, which contains a circularly permuted green fluorescent protein domain, was introduced to monitor the intracellular Ca^2+^ influx in response to NO‐mediated activation of TRPV1 (Figure 5a).

Consistent with previous findings, we found that TRPV1‐expressing cells (TRPV1^+^) can be triggered by the NO.^[^ ^45^ ^]^ Following the addition of 10 mM of the NO donor 2‐(*N*,*N*‐diethylamino) diazenolate‐2‐oxide (DEA NONOate), 75.3% of TRPV1^+^ showed a significant increase in intracellular Ca^2+^ (as marked by the normalized GCaMP6s fluorescence increase >50%). In contrast, cells not expressing TRPV1 (TRPV1^−^) showed no noticeable Ca^2+^ responses at the identical NO donor concentration, and 10 mM of the NH_3_ donor NH_4_Cl did not trigger Ca^2+^ influx into the TRPV1^+^ cells (Figure S8). These results indicated that TRPV1^+^ cells can be activated by NO, not by NH_3_.

We next evaluated whether our electrocatalytic system can control NO‐mediated signaling pathways. For these experiments, carbon electrodes loaded with Cu_2_MoS_4_ or FeCuMoS_4_ electrocatalysts served as the cathode, while a Pt electrode and an Ag/AgCl electrode were utilized as the anode and reference electrodes, respectively. Here, electrocatalyst‐loaded cathodes were fabricated by drop‐casting an ink containing electrocatalysts and a Nafion binder onto oxygen‐functionalized carbon paper, with a catalyst loading of 0.39 mg cm^−2^. TRPV1^+^ cells were placed in close proximity to the cathodes where the NO_2_^−^ reduction reactions occur. Before and during the electrolysis in Tyrode's solution containing NaNO_2_, NO‐mediated activation of TRPV1^+^ cells was recorded with an inverted fluorescent microscope (Figure 5b). Based on product quantification results (Figure 3f–i), we selected an applied voltage of −1.2 V versus Ag/AgCl, at which Cu_2_MoS_4_ and FeCuMoS_4_ exhibit the greatest difference in product selectivity toward NH_3_ and NO (Table S3).

Cu_2_MoS_4_ electrocatalysts, which selectively produce NH_3_ from NO_2_^−^ at an applied voltage of −1.2 V versus Ag/AgCl (Figure 3f), did not trigger TRPV1. In this condition, only 0.7% of TRPV1^+^ showed noticeable Ca^2+^ responses (as marked by the normalized GCaMP6s fluorescence increase >50%) (Figure 5c). In contrast, FeCuMoS_4_ electrocatalysts, which predominantly generate NO from NO_2_^−^ at the identical reaction condition (Figure 3i), induced strong intracellular Ca^2+^ influx in TRPV1^+^ cells. TRPV1^+^ cells began to activate after 386  ±  55 s of electrolysis, and 72.7% of TRPV1^+^ cells showed robust Ca^2+^ responses (Figure 5d). Time‐lapse images showed that TRPV1^+^ cells located in the vicinity of the electrode responded first, and further electrosynthesis activated TRPV1 at a greater distance from the electrode, reflecting the diffusion‐driven gradient of NO extending from the electrode surface (Figure 5e,f). No noticeable Ca^2+^ influx was observed in TRPV1^−^ cells in the presence of NO_2_^−^ ions nor in TRPV1^+^ cells in the absence of NO_2_^−^ ions, indicating that the observed Ca^2+^ responses originated from the interaction between TRPV1 and NO produced by FeCuMoS_4_ electrocatalysts (Figures S9 and S10).

We then next tested whether our system can be similarly utilized to control the NH_3_‐mediated cell signaling pathways. It has been well‐known that NH_3_ gaseous molecules in equilibrium with NH_4_^+^ ions can cross the cell membrane, and these NH_3_ molecules inside the cytosol can alkalinize the intracellular space by uptaking H^+^ ions.^[^ ^46^, ^47^ ^]^ The effect of NH_3_ to alkalinize the intracellular space can create the driving force for the proton influx inside the cell, triggering proton‐selective ion channels.^[^ ^48^ ^]^ Among diverse sets of pH‐sensing ion channels, we chose otopetrin 1 (OTOP1) ion channels, which are broadly expressed in the nervous and gustatory systems, as our testbeds.^[^ ^15^, ^49^ ^]^ Recent studies showed that OTOP1 could be the major ion channel to sense NH_3_ in biological systems.^[^ ^15^ ^]^

**Figure 6:**
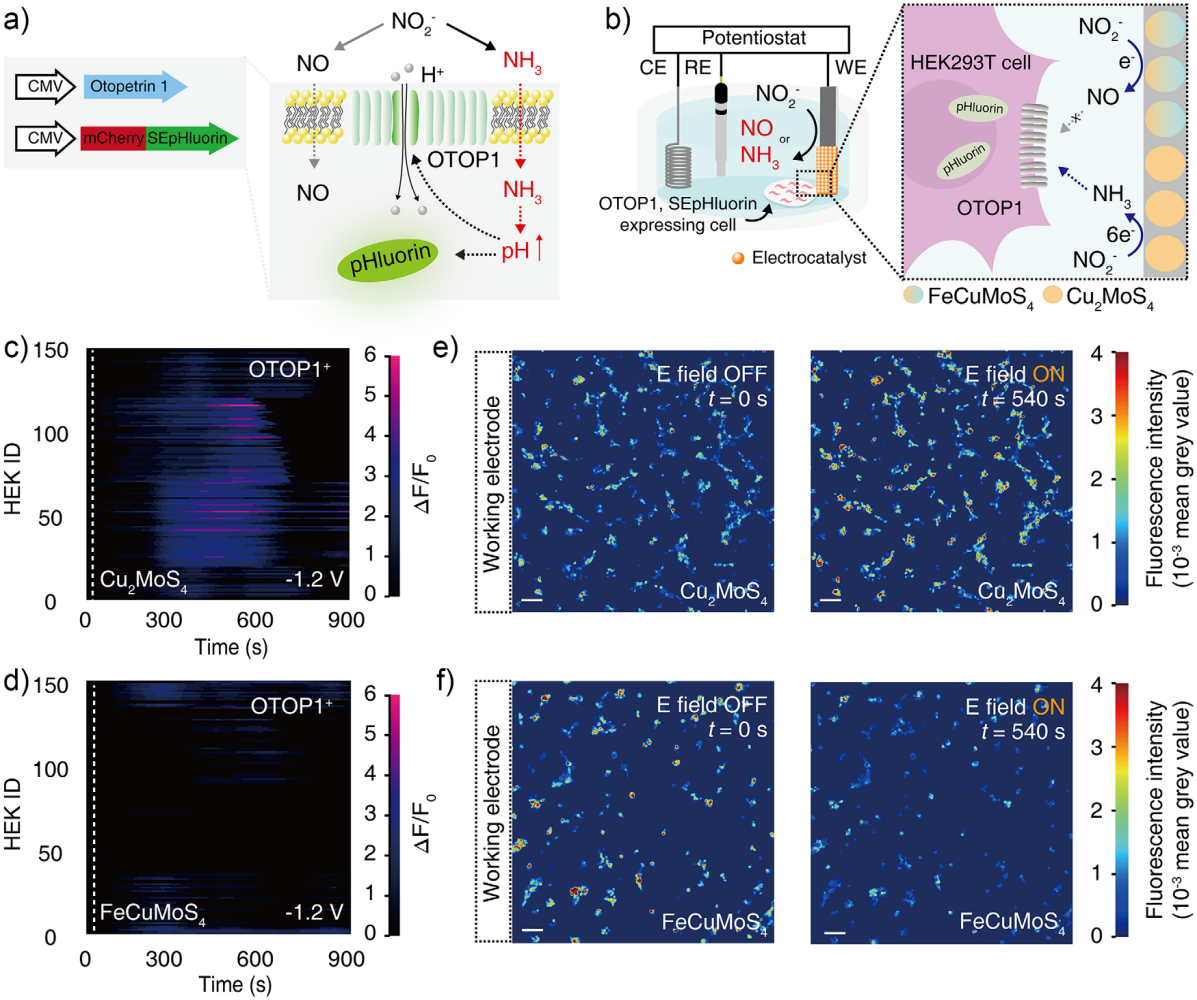
OTOP1 activation mediated by electrosynthesized NH_3_. a) Schematic of plasmids used to prepare cells co‐expressing OTOP1 and SEpHluorin (left) and the mechanism of NH_3_‐mediated intracellular alkalinization and consequent activation of OTOP1 (right). b) Schematic illustrating selective intracellular alkalinization by electrochemically produced NH_3_, but not by NO. Here, cells were positioned near the electrode where NO or NH_3_ signaling molecules were locally produced. Individual SEpHluorin fluorescence traces for 150 OTOP1^+^ cells following the electrosynthesis mediated by c) Cu_2_MoS_4_ and d) FeCuMoS_4_ electrocatalysts. −1.2 V versus Ag/AgCl was applied from 30 s (dashed lines) to 900 s. Here, HEK ID refers to individual cells, with each horizontal line representing the SEpHluorin fluorescence changes of the individual cell over time. For each condition, fluorescence responses were recorded from a total of 150 cells (*n* = 3 independent experiments per group). Time‐lapse images of OTOP1^+^ cells, which were placed in the vicinity of the electrode, upon applying voltages of −1.2 V versus Ag/AgCl to e) Cu_2_MoS_4_ or f) FeCuMoS_4_ electrocatalysts (scale bar: 100 µm). The vertical axis represents SEpHluorin fluorescence intensity, and the color scale was employed to visualize the SEpHluorin fluorescence changes.

To see whether NH_3_ can indeed alkalinize cell cytosol and activate OTOP1, HEK cells were co‐transfected with two plasmids: one encoding mouse OTOP1 under the broad cytomegalovirus promoter (CMV::mOTOP1) and another encoding the genetically encoded fluorescent pH indicator SEpHluorin linked to the pH‐insensitive, fluorescent protein mCherry under the broad cytomegalovirus promoter (CMV::SEpHluorin‐mCherry). The latter was used to measure the intracellular pH changes as a proxy for the OTOP1 currents evoked by NH_3_ (Figure 6a).^[^ ^15^ ^]^

Akin to prior research, the introduction of 10 mM of NH_4_Cl induced noticeable increases in intracellular pH in OTOP1‐expressing (OTOP1^+^) cells (as marked by the normalized SEpHluorin fluorescence increase >30%).^[^ ^46^, ^47^ ^]^ Application of the 10 mM DEA NONOate similarly alkalinized OTOP1^+^ cells; however, these results may have originated from diethylammonium ions, NH_2_(C_2_H_5_)_2_^+^, in the DEA NONOate, whose basic form NH(C_2_H_5_)_2_ could uptake intracellular protons similar to NH_3_. Indeed, an increase in intracellular pH in OTOP1^+^ cells was observed in control experiments using NH_2_(C_2_H_5_)_2_^+^ ions without NO (Figure S11). These results confirmed that intracellular alkalinization and subsequent activation of OTOP1 could be achieved by NH_3_, not NO.

We then applied our electrocatalytic system to modulate NH_3_‐mediated signaling pathways. For these experiments, we employed an identical electrocatalytic setup, with Cu_2_MoS_4_‐ or FeCuMoS_4_‐loaded cathodes, Pt anode, and Ag/AgCl reference, but replaced TRPV1^+^ cells with OTOP1^+^ cells (Figure 6b). The application of −1.2 V versus Ag/AgCl to the Cu_2_MoS_4_ electrocatalysts produced NH_3_ from NO_2_^−^ (Figure 3f), followed by noticeable increases in intracellular pH of OTOP1^+^ cells (Figure 6c). 72.7% of OTOP1^+^ cells showed noticeable pH changes (as marked by the normalized SEpHluorin fluorescence increase >30%). In contrast, the application of the identical voltages to the FeCuMoS_4_ electrocatalysts did not alkalinize the cytosol of OTOP1^+^ cells due to the predominant synthesis of NO (Figure 6d). Time‐lapse images clearly showed that Cu_2_MoS_4_ electrocatalysts induced SEpHluorin fluorescence increases over time, indicating NH_3_‐mediated intracellular alkalinization, whereas FeCuMoS_4_ resulted in minimal changes in SEpHluorin fluorescence (Figure 6e,f). Control experiments using Tyrode's solution without NaNO_2_ precursors demonstrated that Cu_2_MoS_4_ electrocatalysts did not change the intracellular pH of OTOP1^+^ cells at the voltage of −1.2 V versus Ag/AgCl, confirming that observed intracellular alkalinization in OTOP1^+^ cells resulted from electrosynthesized NH_3_ (Figure S12). Together, these results demonstrated that our bioelectrosynthesis platform can selectively regulate NO‐ or NH_3_‐mediated signaling pathways depending on the type of electrocatalyst used.

Note that these experiments were performed without changing the precursor type, concentration, or applied voltages. While the full multiplexing capability of our system has not yet been demonstrated by simultaneously exposing two cell populations (TRPV1^+^ and OTOP1^+^ cells) to both electrocatalysts within a single system, this demonstration highlights its feasibility to independently control dual signaling pathways, which is an important capability for potential in vivo applications. Considering that TRPV1 and OTOP1 ion channels are widely expressed across multiple organ systems, including the dorsal root ganglia and taste buds, our platform holds promise for modulating complex physiological processes in these organs.^[^ ^49^, ^50^, ^51^, ^52^, ^53^, ^54^ ^]^ Additionally, this system could be extended to regulate other biologically relevant signaling pathways involving NO and NH_3_, such as neurotransmission, vascular homeostasis, and inflammatory responses.^[^ ^21^, ^55^, ^56^, ^57^, ^58^ ^]^

Besides its capability to control dual signaling pathways, we found that our electrocatalytic system enables dynamic and precise regulation of cellular signaling through tunable electrochemical inputs. Numerical simulations provided insights into how the release kinetics and concentration profiles of NO and NH_3_ within the cellular microenvironments can be tuned by adjusting the applied voltage. Specifically, the simulations showed that more negative voltages lead to higher local concentrations of signaling molecules near the electrode surface, with concentrations decreasing over distance due to diffusion (Figures S13 and S14). The simulated voltage‐dependent concentration gradients align with the experimentally observed cellular activation dynamics. Experimentally, more negative voltages accelerated the onset of TRPV1^+^ or OTOP1^+^ cell activation, as evidenced by earlier Ca^2+^ influx or intracellular alkalinization under −1.4 V compared to −1.2 V (Figures S15 and S16). Together, these results demonstrate that our system can modulate the activation timing of ion channels and downstream signaling pathways through voltage control.

We further showed that another electrochemical input, electrolysis duration, determines the spatial extent of cellular activation. As shown in Figure 5f, cells are activated in a distance‐dependent manner as NO and NH_3_ diffuse outward from the electrode. By leveraging this phenomenon, we precisely controlled the stimulation area by varying the duration of electrolysis (Figures S17 and S18). Additionally, we demonstrated that the modulation of another electrochemical input, applied voltage waveform, can enable the on‐demand termination of cellular signaling. For example, when a step‐function‐like voltage waveform was employed by applying −1.2 V to the FeCuMoS_4_ electrocatalysts and then abruptly switching off the voltage, electrosynthesis of NO terminated immediately, preventing the further activation of TRPV1^+^ cells (Figure S19).

Together, these findings confirm that electrochemical inputs in our system can be directly translated into biological outputs. Specifically, we revealed that electrochemical parameters, such as applied voltage, electrolysis duration, and voltage waveform, can be used to modulate key aspects of cellular responses, including activation timing, spatial stimulation range, and termination point of cellular activation. The ability to regulate cellular responses with high spatiotemporal precision offers distinct advantages over conventional donor‐based approaches (Figures S17 and S18). Given this unique capability, our platform may also be extended beyond ion channel regulation to modulate more complex biological responses, such as gene expression or controlled protein secretion, within the context of electrogenetics.^[^ ^59^, ^60^, ^61^, ^62^ ^]^

## Conclusion

In conclusion, we have developed a bioelectrosynthesis platform capable of selectively generating two distinct signaling molecules, NO and NH_3_, and delivering them to targeted cells. By leveraging enzyme‐inspired Cu_2_MoS_4_ and FeCuMoS_4_ electrocatalysts, we demonstrated that multiple signaling molecules can be selectively synthesized from a single precursor, NO_2_^−^ ions, directly within biological systems. Computational studies suggested that the differing selectivity of Cu_2_MoS_4_ and FeCuMoS_4_ for NO and NH_3_ predominantly arises from their distinct binding affinities for NO intermediates. Our bioelectrosynthesis platform, using these electrocatalysts, enabled targeted control of NO‐ or NH_3_‐mediated cell signaling processes by activating NO‐sensitive (TRPV1) or NH_3_‐sensitive (OTOP1) ion channels expressed in human cell lines. We envision that this platform may serve as a tool for investigating and controlling complex signaling processes involving NO and NH_3_.

## Supporting Information

The authors have cited additional references within the Supporting Information.^[^ ^63^, ^64^, ^65^, ^66^, ^67^, ^68^, ^69^, ^70^, ^71^, ^72^, ^73^ ^]^

## Conflict of Interests

The authors declare no conflict of interest.

## Supporting information



Supporting Information

## Data Availability

The data that support the findings of this study are available from the corresponding author upon reasonable request.
