# Supplementary material for: Bioelectrosynthesis of Signaling Molecules for Selective Modulation of Cell Signaling
Source: Angew Chem Int Ed Engl. 2025 Aug 4;64(37):e202508192. doi: 10.1002/anie.202508192 (PMC12416457; doi:10.1002/anie.202508192)
Supplement: Supplementary file 1 — Supporting Information [file ANIE-64-e202508192-s001.docx]

**Supplementary Information**

**Bioelectrosynthesis of Signaling Molecules for Selective Modulation of Cell Signaling**

Myeongeun Lee^†[a]^, Jaewoong Lee^†[a]^, Yongha Kim^[a]^, Changho Lee^[a]^, Sang Yeon Oh^[a]^, Jihan Kim^[a]^, Jimin Park*^[a]^

[a] M. Lee, J. Lee, Y. Kim, C. Lee, S. Y. Oh, Prof. J. Kim, Prof. J. Park*

Department of Chemical and Biomolecular Engineering

Korea Advanced Institute of Science and Technology (KAIST)

Daejeon 34141, South Korea

E-mail: [jiminp@kaist.ac.kr](mailto:jiminp@kaist.ac.kr)

[^†^] These authors contributed equally to this work

**Methods**

**Materials**

Copper(I) oxide (Cu_2_O, powder, ≤7 μm, 97%), ammonium tetrathiomolybdate ((NH_4_)_2_MoS_4_, 99.97% trace metals basis), sodium molybdate dihydrate (Na_2_MoO_4_∙2H_2_O, ACS reagent, ≥99%), copper(II) acetate monohydrate (Cu(CO_2_CH_3_)_2_∙H_2_O, ACS reagent, ≥99%), L-cysteine (97%), iron(II) acetate (Fe(CO_2_CH_3_)_2_, ≥99.99% trace metals basis), ethylene glycol (ReagentPlus®, ≥99%), ethyl alcohol (≥99.5%, ACS reagent, 200 proof), 1-propanol (ACS reagent, ≥99.5%), dimethyl sulfoxide (DMSO, anhydrous, ≥99.9%), sodium nitrite (ACS reagent, ≥97.0%), ammonium chloride (ACS reagent, ≥99.5%), sodium salicylate (C_6_H_4_(COONa)(OH), ReagentPlus®, ≥ 99.5%), sodium hypochlorite (NaOCl, reagent grade, 10-15% available chlorine), sodium hydroxide (NaOH, ACS reagent, ≥97.0%, pellets), sodium nitroprusside (Na_3_[Fe(CN)_5_(NO)]·2H_2_O, ACS reagent, ≥99%), trichloroacetic acid (ACS reagent, ≥99.0%), sodium carbonate (powder, ≥99.5%, ACS reagent), hydroxylamine solution (50 wt% in H_2_O, 99.999%), sulfamic acid (ACS reagent, 99.3%) and 8-quinolinol (ACS reagent, ≥ 99%) were purchased from Sigma Aldrich. Propylamine propylamine NONOate (PAPA NONOate, Purity > 97%) and diethylamine NONOate (DEA NONOate, Purity 97%) were purchased from Abcam. 4-amino-5-methylamino-2',7'-difluorofluorescein, (DAF-FM) and Nafion® D-520 dispersion (5% w/w in water and 1-propanol) were purchased from Thermo Fisher Scientific. Tyrode's solution consisted of 125 mM sodium chloride (NaCl, ≥ 99 % Sigma Aldrich), 2 mM potassium chloride (KCl, ≥99 %, Sigma Aldrich), 2 mM magnesium chloride hexahydrate (MgCl_2_·6H_2_O, ≥ 99 %, Sigma Aldrich), 2 mM calcium chloride dihydrate (CaCl_2_·2H_2_O, ≥ 99%, Sigma Aldrich), 25 mM 4-(2-hydroxyethyl)-1-piperazineethanesulfonic acid (HEPES, ≥ 99.5 %, Sigma Aldrich), and 51 mM D-glucose (≥ 99.5%, Sigma Aldrich). The pH was carefully adjusted to 7.4 using NaOH solution (1 M).

**Synthesis of Cu_2_MoS_4_ and FeCuMoS_4_ electrocatalysts**

Cu_2_MoS_4_ was synthesized using a solvothermal method.^[36]^ Specifically, 0.1371 g of Cu_2_O and 0.3702 g of (NH_4_)_2_MoS_4_ were dissolved in 80 mL of ethylene glycol. This solution was sonicated for 30 min, followed by stirring for 1 h. The prepared solution was then transferred to a 100 mL autoclave and heated at 190 °C for 12 h. After naturally cooling to room temperature, the resultant product was centrifuged at 11,000 rpm for 10 min and then washed three times with deionized water and ethanol to remove residual impurities. After the washing process, the product was dried at 60 °C overnight. For the synthesis of FeCuMoS_4_, 0.24195 g of Na_2_MoO_4_∙2H_2_O, 0.19965 g of Cu(C_2_H_3_O_2_)_2_∙H_2_O, 1.2116 g of L-cysteine, and 0.17393 g of Fe(C_2_H_3_O_2_)_2_ were dissolved in deionized water.^[59]^ This mixture was heated at 180 °C for 14 h and then purified with the method used for Cu_2_MoS_4_.

**Characterization of Cu_2_MoS_4_ and FeCuMoS_4_ electrocatalysts**

The phase of the synthesized Cu_2_MoS_4_ and FeCuMoS_4_ was determined using powder XRD on a high-resolution diffractometer (SmartLab, 9 kW) with Cu Kα radiation (λ = 1.54056 Å). The surface morphology and structural characteristics of Cu_2_MoS_4_ and FeCuMoS_4_ were analyzed with a field-emission SEM (Magellan 400, FEI). HRTEM and STEM images of Cu_2_MoS_4_ and FeCuMoS_4_ were obtained with a multi-EDS equipped field-emission TEM (Talos F200X G2, Thermo Fisher, 200 kV). The atomic composition of Cu_2_MoS_4_ and FeCuMoS_4_ was quantified using ICP-OES (Agilent 5110).

**Electrode preparation and electrochemical study**

For chronoamperometric measurements, carbon paper (Toray, TGP-H-60, 5 wt% wet-proofed) with a surface area of 4 cm² was thermally treated in a box furnace at 800 °C in static air for 10 min to produce oxygen-functionalized carbon paper (OxCP). Catalyst ink was prepared by dispersing 15 mg of Cu_2_MoS_4_ or FeCuMoS_4_ in 2025 μL of a 0.123 wt% Nafion solution (Nafion D-520, 5 wt%, Thermo Fisher Scientific), which was subsequently sonicated for 1 h. The Nafion solution was diluted with ethanol or 1-propanol to ensure adequate dispersion. The cathode was fabricated by drop-casting 71 μL of freshly sonicated ink onto the OxCP. The droplet rapidly spread across the carbon paper, ensuring uniform coverage, and the electrode was then air-dried in an oven at 60 °C for 1 h. This drop-casting and drying process was repeated twice more, resulting in a total of 213 μL of catalyst ink deposited on each electrode. For CV measurements, 5 μL of the catalyst ink was applied onto a freshly cleaned glassy carbon electrode (diameter 3 mm, model 013714, ALS). Pt coil (012961, ALS) was used as the anode and Ag/AgCl electrode (RE-1B, 012167, ALS) was used as the reference electrode. The electrochemical measurements were conducted with Interface 1010E (Potentiostat, Gamry).

**NO, NH_4_^+^ and NH_2_OH quantification**

To quantify NO, an external calibration curve was generated using PAPA NONOate as the NO donor. PAPA NONOate releases approximately 1.5 equivalents of NO with a half-life of 15 min at 37 °C and pH 7.4.^[60,61]^ For constructing the calibration curve, 100 μL of PAPA NONOate solutions, with concentrations spanning from 0 to 600 μM, were mixed with 100 μL of 600 μM DAF-FM in Tyrode's solution in a 96-well plate.^[62]^ Fluorescence intensity for each mixture of PAPA NONOate and DAF-FM was recorded over a 16 h period until an equilibrium state was achieved. For electrochemically generated NO, DAF-FM in Tyrode's solution was immediately added to the electrolyte post-electrolysis to allow direct, fluorescence-based quantification of NO within the electrochemical system.

For the quantification of ammonium (NH_4_^+^) in a 0.1 M NaNO_2_ Tyrode's solution, a salicylate-based colorimetric method was applied.^[63]^ The reagent solution was composed of 2.5 M sodium salicylate and 8 mM sodium nitroprusside as the primary components. A 1% hypochlorite solution was also prepared by mixing 0.4 M NaOH with a 10–15% sodium hypochlorite solution. Following electrolysis, 280 μL of the salicylate solution and 280 μL of the 1% hypochlorite solution were introduced to the electrolyte. The mixture was then transferred to a 96-well plate for UV-Vis spectroscopic analysis, with a calibration curve established using NH_4_^+^ standards at 0, 250, 500, and 1000 μM concentrations.

To detect NH_2_OH, the indooxine dye method was employed.^[39,41]^ This assay involves the reaction of NH_2_OH with specific colorimetric reagents to yield the green indooxine dye. The assay solution comprised 12 wt% trichloroacetic acid, 0.07 M 8-quinolinol in ethanol, 0.05 M phosphate buffer, deionized water, and 1 M sodium carbonate, which were introduced into the electrolyte post-electrolysis. Prior to reagent addition, the electrolyte was treated with 0.2 M sulfamic acid to eliminate nitrite, as nitrite could interfere with the colorimetric detection by reacting with assay reagents.^[40]^ Upon the addition of all reagents, the solution was heated in boiling water for 1 min, then allowed to cool to room temperature over 30 min. A calibration curve was generated using NH_2_OH standard solutions at concentrations of 0, 250, 500, 1000, and 2000 μM, with absorbance measurements taken at 705 nm via UV-Vis spectrophotometry.

**Computational details**

Electronic structure relaxations, free energy calculations, and Bader charge analyses were performed using the Vienna Ab initio Simulation Package (VASP). The electronic wave functions were expanded in plane waves with a cutoff energy of 520 eV. Convergence criteria were set to 10^-5^ for total energy and 0.01 eV/Å for forces. The Perdew–Burke–Ernzerhof exchange-correlation functional was employed. To calculate free energies, the computational hydrogen electrode model was applied, assuming the chemical potential of an electron–proton pair to be equivalent to that of 1/2 H_2_ in the gas phase. The adsorption free energies (ΔG) were computed as following formula:

∆G = ∆E_DFT_- ∆(ZPE-TS)

where ∆E_DFT_ represents the enthalpy, ZPE is the zero-point energy, and TS denotes the entropic contribution at a given temperature, respectively.

**Cell culture**

The HEK 293T cells were cultured in DMEM (WELGENE, LM001-08) supplemented with 10% FBS (WELGENE, S001-01), 1% penicillin-streptomycin (WELGENE, LS 202-02), and 1% GlutaMAX (Gibco). The HEK 293T cell line was purchased from ATCC and was confirmed to be free from mycoplasma contamination via microscopic examination. Prior to cell seeding, 12 mm diameter glass coverslips (German Glass, Electron Microscopy) in each well of a 24-well plate were coated with 300 µl of Poly-D-Lysine solution (Gibco) for 1 h. For transfection, each well received 1 µl of P3000 reagent (Invitrogen), 1.5 µl of Lipofectamine 3000 (Invitrogen), and 500 ng of plasmid DNA in 50 µl Opti-MEM. AAV-CamKII-TRPV1-P2A-mCherry (Addgene plasmid #200829), pGP-CMV-GCaMP6s (Addgene plasmid #40753), mOtop1_pcDNA3 (Addgene plasmid #114677), and mCherry-SEpHluorin (Addgene plasmid #32001) were used for the transfection. AAV-CamKII-TRPV1-P2A-mCherry was a gift from Hong Chen (Addgene plasmid # 200829 ; http://n2t.net/addgene:200829 ; RRID:Addgene_200829).^[64]^ pGP-CMV-GCaMP6s was a gift from Douglas Kim & GENIE Project (Addgene plasmid # 40753 ; http://n2t.net/addgene:40753 ; RRID:Addgene_40753).^[65]^ mOtop1_pcDNA3 was a gift from Emily Liman (Addgene plasmid # 114677 ; http://n2t.net/addgene:114677 ; RRID:Addgene_114677).^[66]^ mCherry-SEpHluorin was a gift from Sergio Grinstein (Addgene plasmid # 32001 ; http://n2t.net/addgene:32001 ; RRID:Addgene_32001).^[67]^

**Fluorescence analysis and in vitro calcium imaging using GCaMP6s**

Fluorescent imaging experiments were conducted on the transfected cells attached to the coverslips 24 h post-transfection. For experiments involving NO donors and NH_4_Cl, HEK 293T cells grown on glass coverslips were placed in 24-well plates with 500 µl of Tyrode’s solution. GCaMP6s fluorescence changes were captured at 1 frame per second using an inverted fluorescence microscope. Following a 30 s baseline recording, 100 µl of Tyrode’s solution containing 50 mM DEA NONOate or NH_4_Cl was introduced to each well. For in vitro validation of the electrochemical NO delivery system, OxCP electrodes loaded with either Cu₂MoS₄ or FeCuMoS₄ (1 cm × 4 cm), a platinum coil electrode, and an Ag/AgCl reference electrode were positioned within each well of a 6-well plate. After positioning the electrodes, each well was filled with 8 ml of Tyrode’s solution containing 25 mM NaNO₂. HEK 293T cells on glass coverslips were positioned adjacent to the OxCP electrode. Following an initial 30 s recording, the voltage was applied to the OxCP electrodes via an Interface 1010E potentiostat (Gamry). This voltage was maintained until the completion of the calcium imaging experiments. The fluorescence intensity of individual cells was quantified using ImageJ software (version 1.53e; National Institutes of Health, USA), with F_0_ values determined by averaging fluorescence intensities over the first 10 s of recording. Mean ΔF/F_0_ values were calculated from 150 randomly selected HEK 293T cells derived from a minimum of three independent experiments.

**In vitro fluorescence-based imaging assay using SEpHluorin**

Imaging experiments for NO donor and NH_4_Cl injection were conducted under the same conditions as the TRPV1 experiments. For the injection of NH_2_(C_2_H_5_)_2_^+^ ions, the NO donor DEA NONOate was incubated in a 37°C water bath for 15 min to completely remove NO before injection. The in vitro validation of the electrochemical NH_3_ delivery system was also performed under the same conditions as the TRPV1 experiments. Fluorescence intensity analysis was conducted using ImageJ, with statistical processing performed on 150 randomly selected HEK 293T cells from a minimum of three independent experiments.

**Numerical simulation methods for the diffusion profiles of NO and NH₃**

The diffusion profiles of NO and NH_3_ were calculated using a simple diffusion reaction model based on the previous report.^[3]^ In this model, we considered the autoxidation process of NO,^[42-43]^ which depletes the electrochemically produced NO according to the equation (1):

$4\mathrm{NO}_{(aq)}+O_{2(g)}+2H_{2}O \leftrightarrow4H^{+}+ 4NO_{2}^{-}$ (1)

The rate law describing the autoxidation process is given by equation (2):

$\frac{\partial C_{\mathrm{NO}}}{\partial t}= -4k_{1}C_{O_{2}}C_{\mathrm{NO}}^{2}$ (2)

Here, an initial dissolved oxygen concentration of 40 mg L⁻¹ (at 1 bar and 25 °C) was used, and its consumption via autoxidation was included in the model.

By combining the electrochemical formation rate of NO with its depletion rate via autooxidation reaction, the spatiotemporal evolution of the NO concentration can be described by the following equation (3):

$\frac{\partial C_{\mathrm{NO}}}{\partial t}=D_{\mathrm{NO}}\frac{\partial^{2}C_{\mathrm{NO}}}{\partial x^{2}}-4k_{1}C_{O_{2}}C_{\mathrm{NO}}^{2}$ (3)

Boundary and initial conditions to solve equation 3 are summarized in equations (4-6):

$C_{\mathrm{NO}}\left( x,t=0 \right)=0$ (4)

$C_{\mathrm{NO}}\left( 50,000\mu m, t \right)=0$ (5)

$D_{\mathrm{NO}}\left. \frac{\partial C_{\mathrm{NO}}}{\partial x} \right|_{x=0}= -\frac{i_{\mathrm{NO}}}{F}$ (6)

In the case of NH_3_, we only considered the formation of NH_3_ at the electrode, as presented in equation (7):

$\frac{\partial C_{NH_{3}}}{\partial t}=D_{\mathrm{NH}_{3}}\frac{\partial^{2}C_{\mathrm{NH}_{3}}}{\partial x^{2}}$ (7)

Boundary and initial conditions to solve equation 7 are summarized in equations (8-10):

$C_{\mathrm{NH}_{3}}\left( x,t=0 \right)=0$ (8)

$C_{\mathrm{NH}_{3}}\left( 50,000\mu m, t \right)=0$ (9)

$D_{\mathrm{NO}}\left. \frac{\partial C_{\mathrm{NH}_{3}}}{\partial x} \right|_{x=0}= -\frac{i_{\mathrm{NH}_{3}}}{6F}$ (10)

where *i*_NO_ and *i*_NH3_ denote the partial current densities corresponding to the formation of NO and NH₃, respectively. The rate constants and diffusion coefficients used to solve the governing equations are as follows: *k*_1_ = 2.9 × 10^6^ M^-2^ s^-1^, D_NO_ = 2.21 × 10^-5^ cm^2^ s^-1^, D_O2_ = 2.5 × 10^-5^ cm^2^ s^-1^, D_NH3_ = 2.05 × 10^-5^ cm^2^ s^-1^ in water at 1 bar and 25 °C.^[3,72-73]^

**Supplementary Figures**


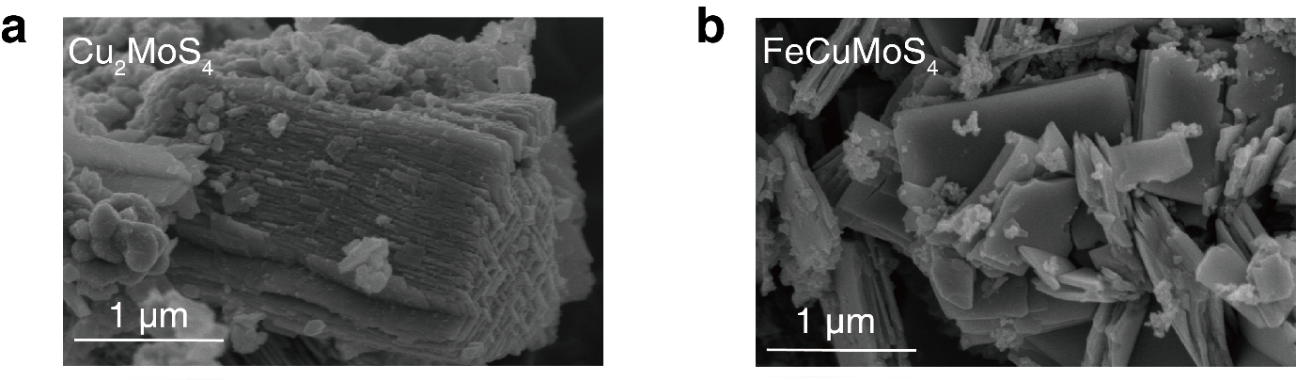


**Figure S1.** SEM images of a) Cu_2_MoS_4_ and b) FeCuMoS_4_ crystals.


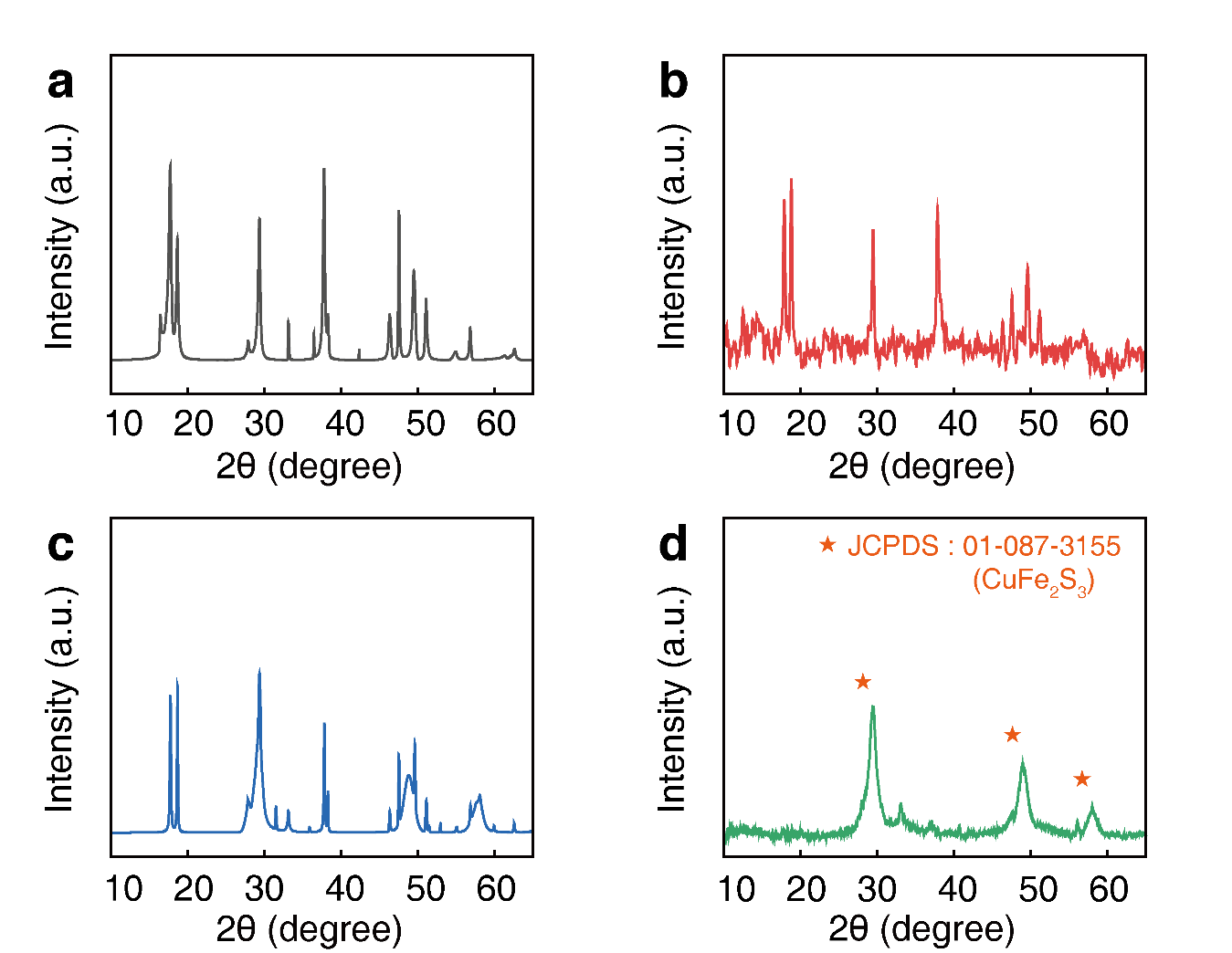


**Figure S2.** XRD patterns of Cu_2_MoS_4_ crystals a) before and b-d) after the Fe doping process with varying Fe doping levels. The molar ratios of the Mo precursor to the Fe precursor are b) 2:1, c) 1:1, and d) 1:2, respectively.


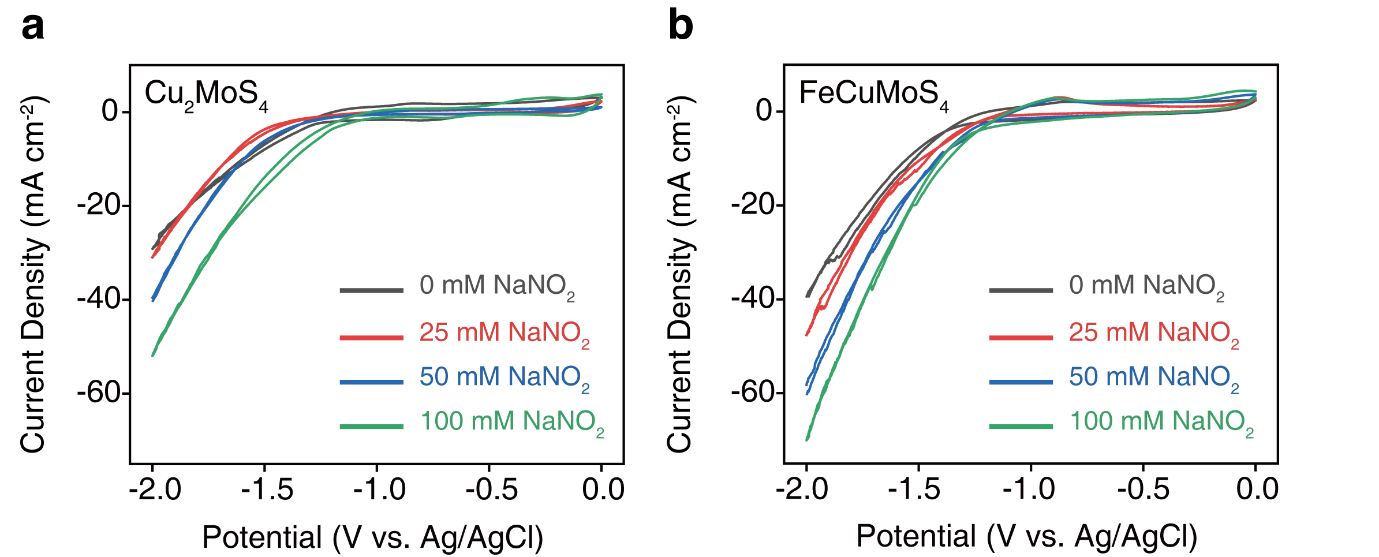


**Figure S3.** CV curves for a) Cu_2_MoS_4_ and b) FeCuMoS_4_ at various NO_2_^-^ concentrations. The overall reduction current increased as NO_2_^-^ concentration increased, confirming that NO_2_^-^ is predominantly involved in the reduction reaction. Note that Faradaic currents appeared in the absence of NO_2_^-^ predominantly resulted from the HER, making direct quantitative comparison between the Faradaic currents in the presence and absence of NO_2_^-^ not feasible.


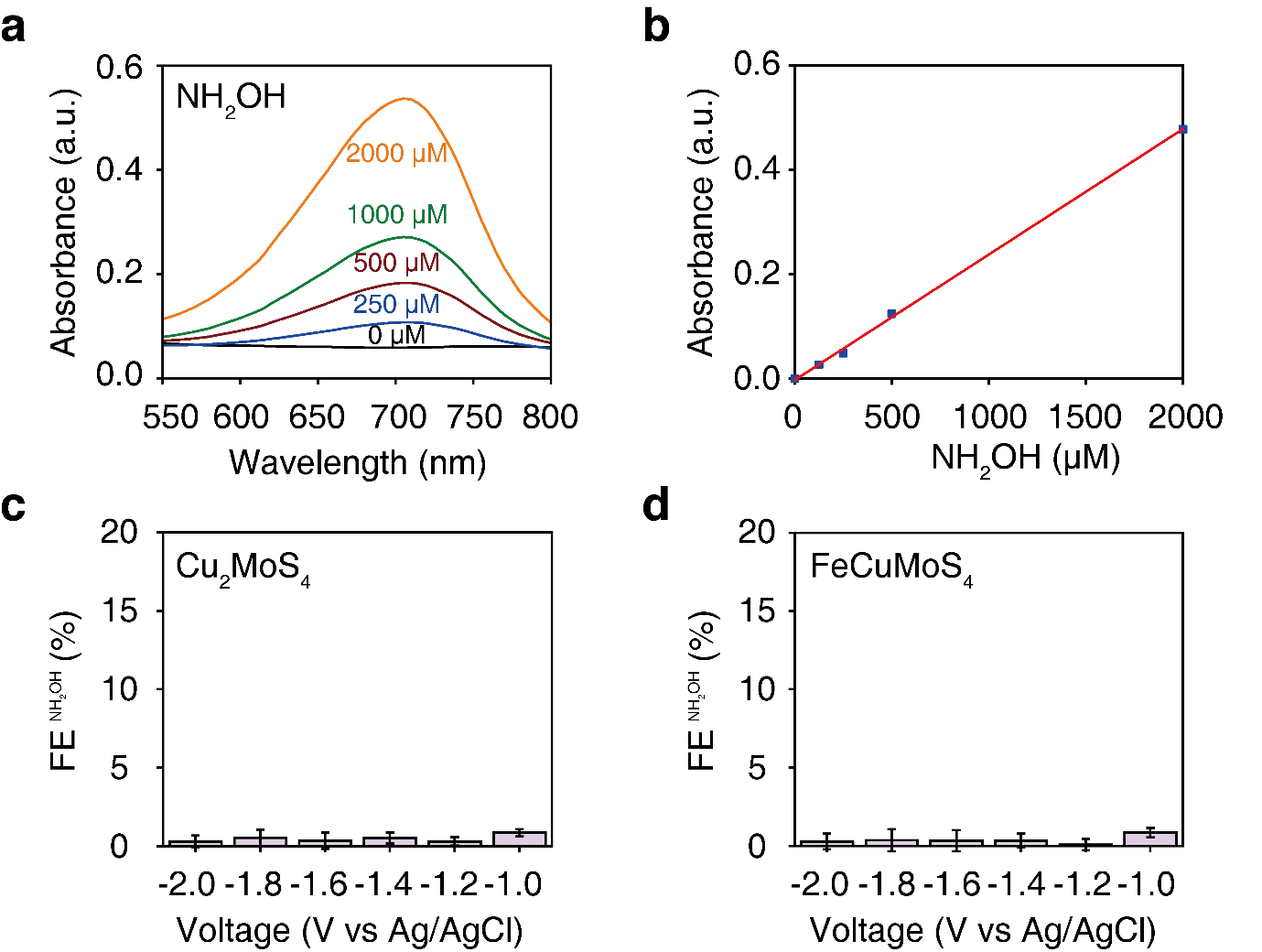


**Figure S4.** Quantification of NH_2_OH. a) Absorption spectra of NH₂OH solutions with concentrations ranging from 0 µM to 2000 µM. b) The calibration curve derived from the absorption at 705 nm from the spectra shown in a). Faradaic efficiency of NH_2_OH (FE_NH2OH_) (mean ± s.d.) with the c) Cu_2_MoS_4_ and d) FeCuMoS_4_ at various applied voltage conditions (n=3 independent experiments per group). A potential range of -1.0 V to -2.0 V versus Ag/AgCl was utilized for the analysis.


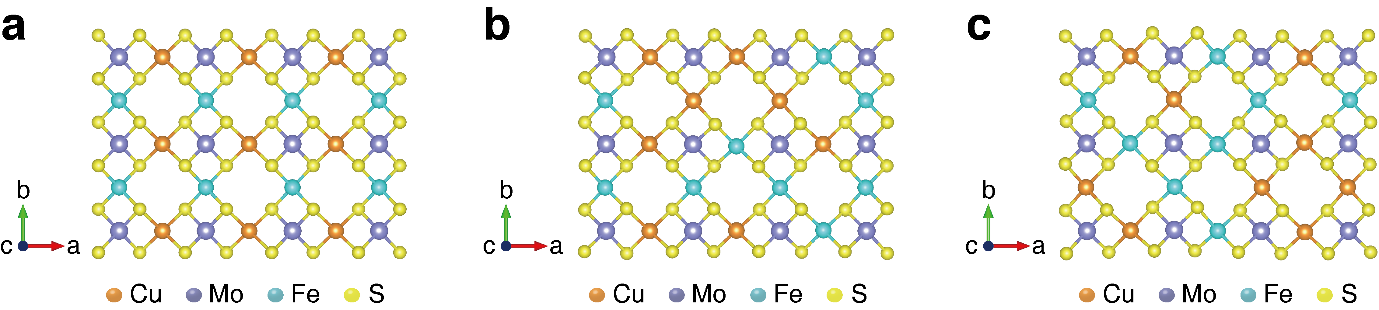


**Figure S5.** a-c) Basal plane configurations of Fe-substituted Cu_2_MoS_4_ models. Based on the experimentally observed Fe:Cu ratio in FeCuMoS₄ (Figure 2g), we constructed FeCuMoS₄ structures by substituting half of the Cu atoms in Cu_2_MoS₄ with Fe. Among the three structures, the most stable configuration a) was selected for the subsequent binding energy and free energy calculations. The energy values are provided in Table S1.


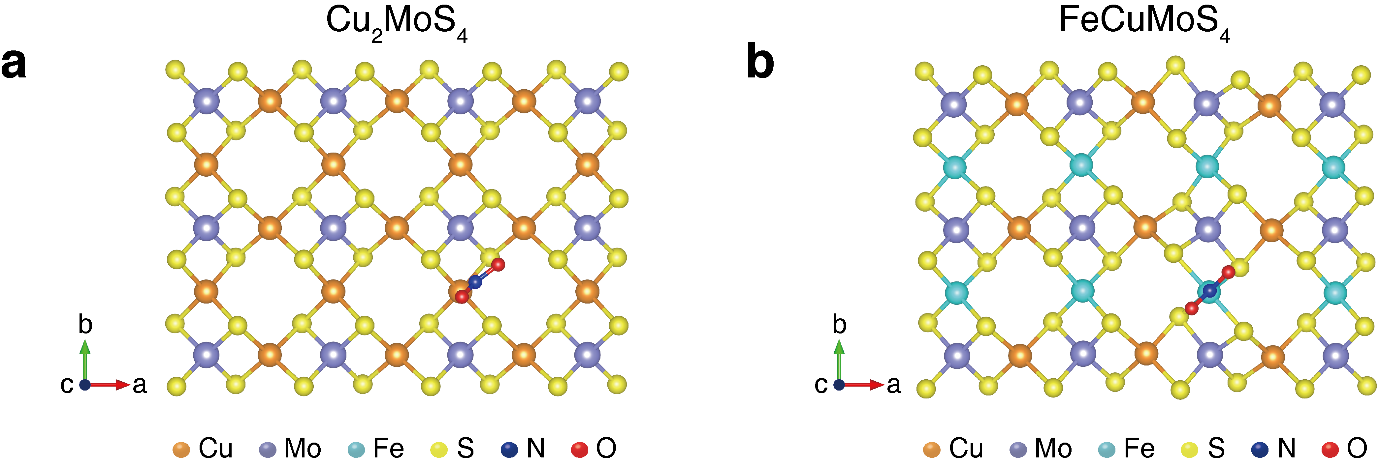


**Figure S6.** Structural representation of NO_2_^-^ bound to the basal planes of a) Cu_2_MoS_4_ and b) FeCuMoS_4_.


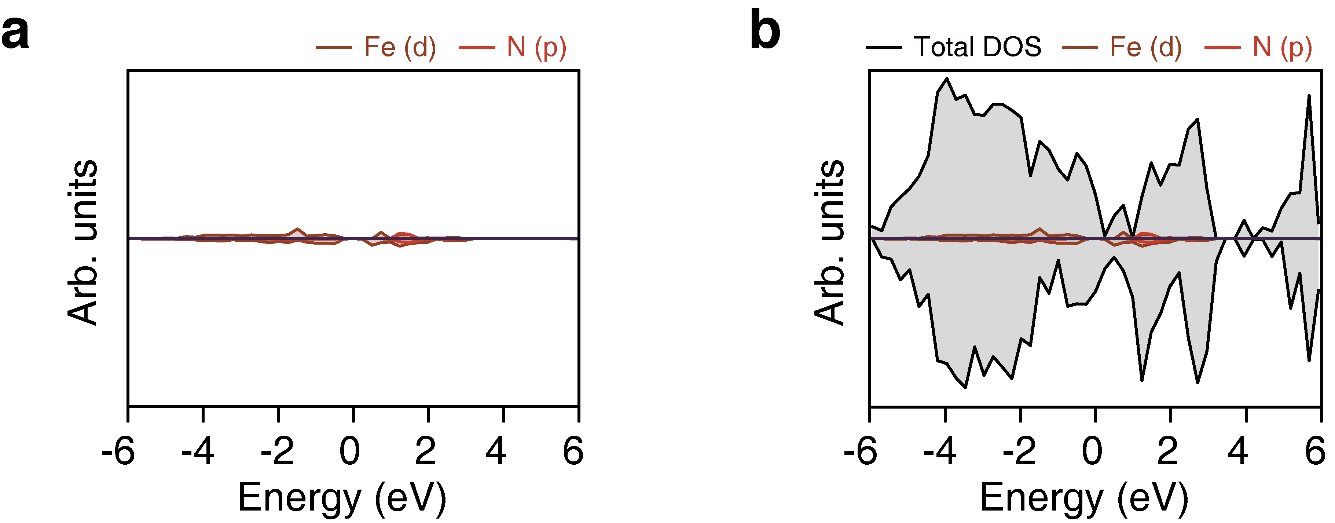


**Figure S7.** DOS for the binding configuration of FeCuMoS_4_ with NO. Visualization of a) the d orbital of Fe and p orbital of N and b) total DOS.


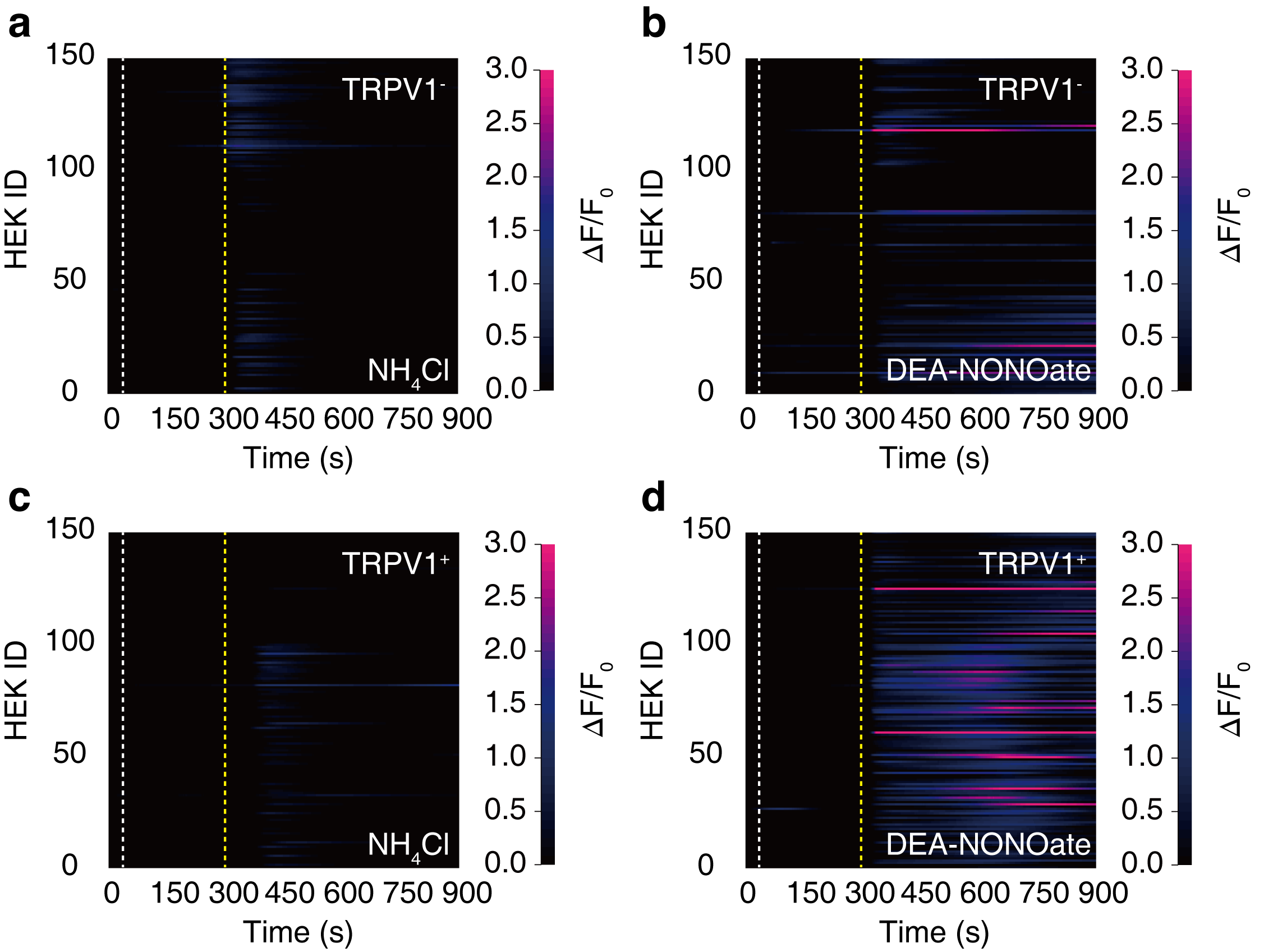


**Figure S8**. Individual GCaMP6s fluorescence traces for 150 TRPV1^-^ cells upon injection of 10 mM a) NH_4_Cl and b) DEA-NONOate. Individual GCaMP6s fluorescence traces for 150 TRPV1^+^ cells upon injection of 10 mM c) NH_4_Cl and d) DEA-NONOate. Tyrode’s solution was injected after 30 s (white dashed lines), and NH_4_Cl (or DEA-NONOate) was injected after 300 s (yellow dashed lines). Tyrode’s solution was utilized to validate the negligible effects of injection procedures on Ca^2+^ influx.


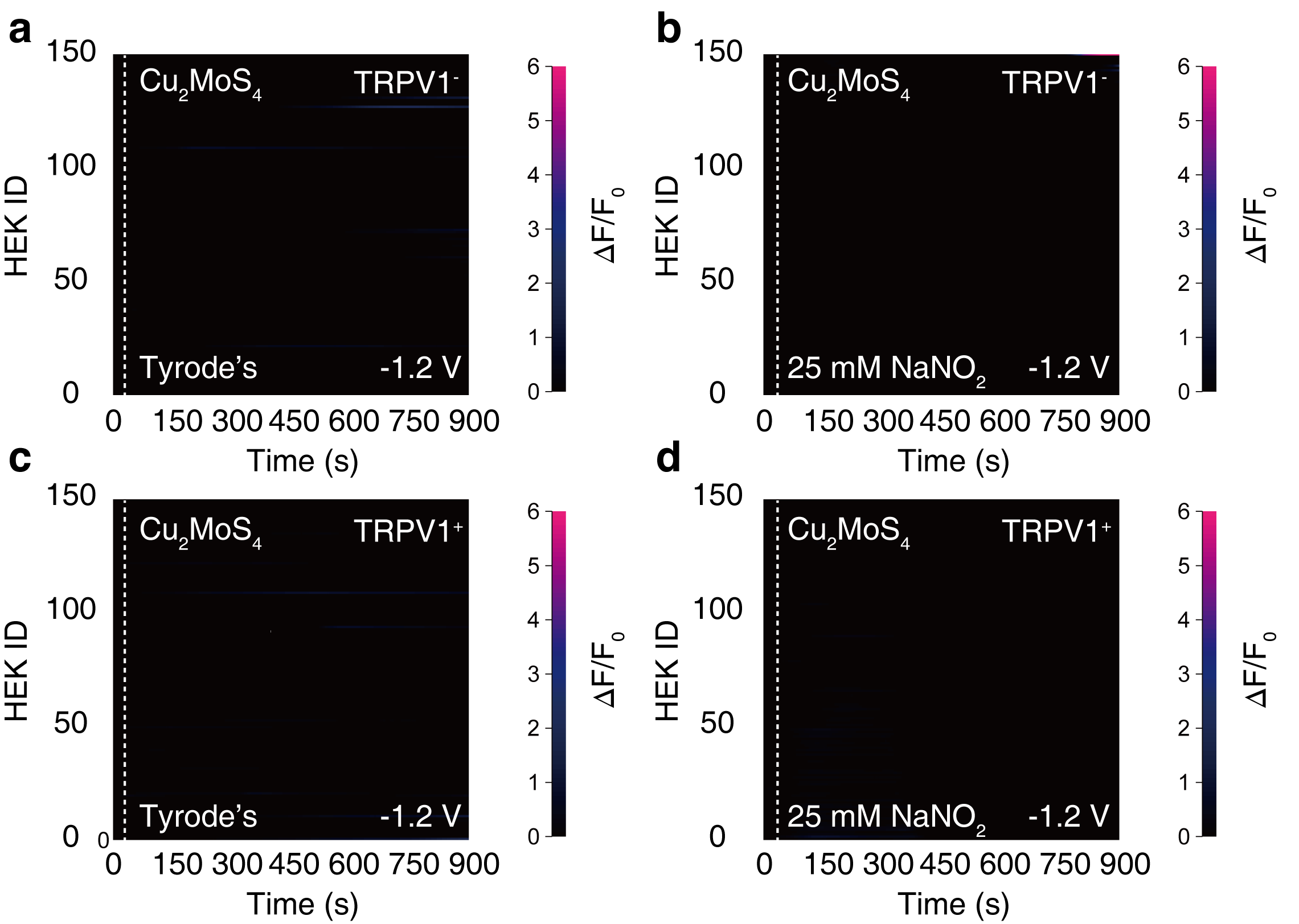


**Figure S9**. Individual GCaMP6s fluorescence traces for 150 TRPV1^-^ cells upon application of a voltage of -1.2 V versus Ag/AgCl to Cu_2_MoS_4_ in the a) absence and b) presence of NO_2_^-^ ions. Individual GCaMP6s fluorescence traces for 150 TRPV1^+^ cells upon application of a voltage of -1.2 V versus Ag/AgCl to Cu_2_MoS_4_ in the c) absence and d) presence of NO_2_^-^ ions. The voltages were turned on 30 s (dashed lines), and continuous electrochemical synthesis was performed until 900 s.


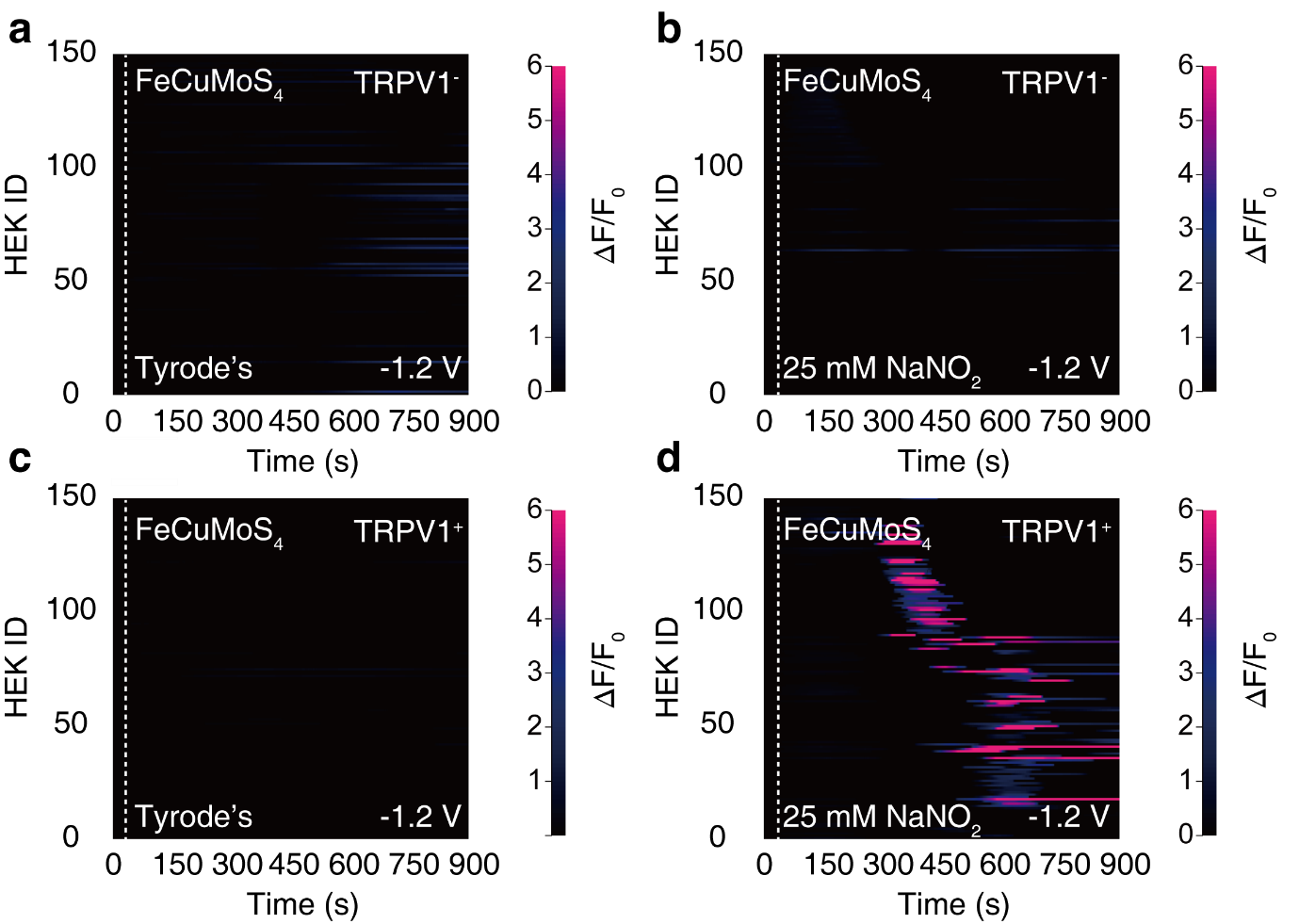


**Figure S10**. Individual GCaMP6s fluorescence traces for 150 TRPV1^-^ cells upon application of a voltage of -1.2 V versus Ag/AgCl to FeCuMoS_4_ in the a) absence and b) presence of NO_2_^-^ ions. Individual GCaMP6s fluorescence traces for 150 TRPV1^+^ cells upon application of a voltage of -1.2 V versus Ag/AgCl to FeCuMoS_4_ in the c) absence and d) presence of NO_2_^-^ ions. The voltages were turned on 30 s (dashed lines), and continuous electrochemical synthesis was performed until 900 s.


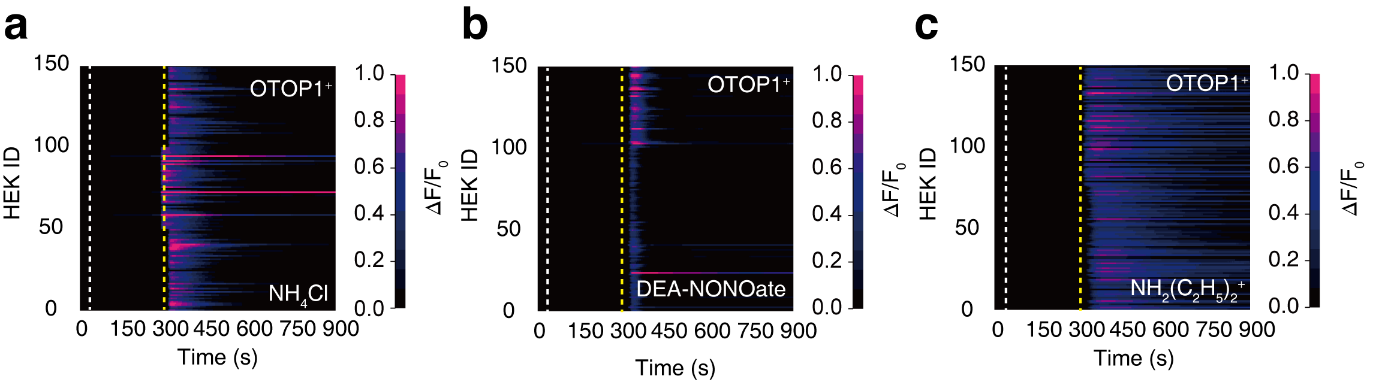


**Figure S11**. Individual SEpHluorin fluorescence traces for 150 OTOP1^+^ cells upon injection of 10 mM a) NH_4_Cl, b) DEA-NONOate and c) NH_2_(C_2_H_5_)_2_^+^ ions. Tyrode’s solution was injected after 30 s (white dashed lines), and NH_4_Cl (or DEA-NONOate or NH_2_(C_2_H_5_)_2_^+^ ions) was injected after 300 s (yellow dashed lines). Tyrode’s solution was utilized to validate the negligible effects of injection procedures on intracellular pH. For the injection of NH₂(C₂H₅)₂⁺ ions, the NO donor DEA NONOate was incubated in a 37°C water bath for 15 min to completely remove NO.


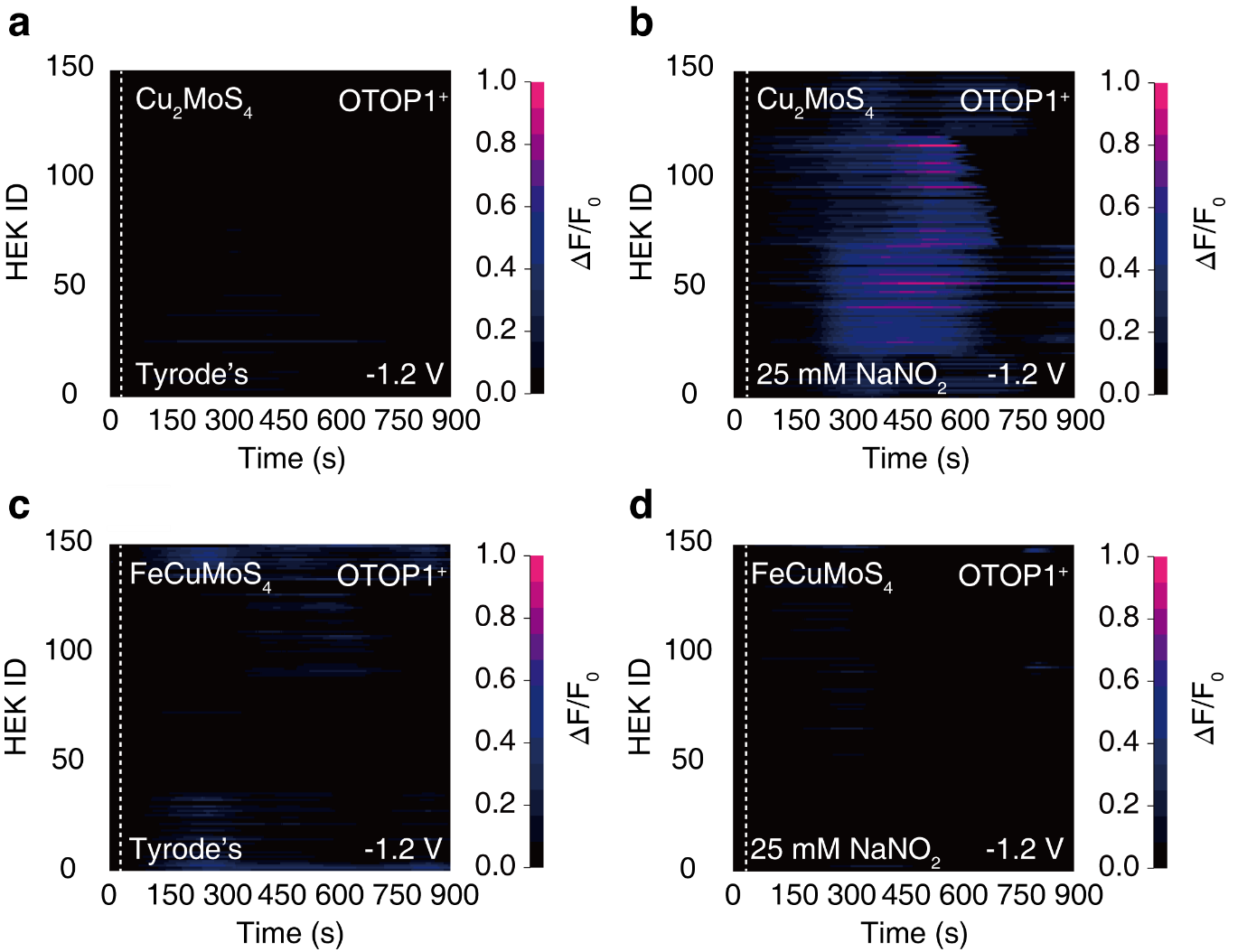


**Figure S12.** Individual SEpHluorin fluorescence traces for 150 OTOP1^+^ cells upon application of a voltage of -1.2 V versus Ag/AgCl to Cu_2_MoS_4_ in the a) absence and b) presence of NO_2_^-^ ions. Individual SEpHluorin fluorescence traces for 150 OTOP1^+^ cells upon application of a voltage of -1.2 V versus Ag/AgCl to FeCuMoS_4_ in the c) absence and d) presence of NO_2_^-^ ions. The voltages were turned on 30 s (dashed lines), and continuous electrochemical synthesis was performed until 900 s.


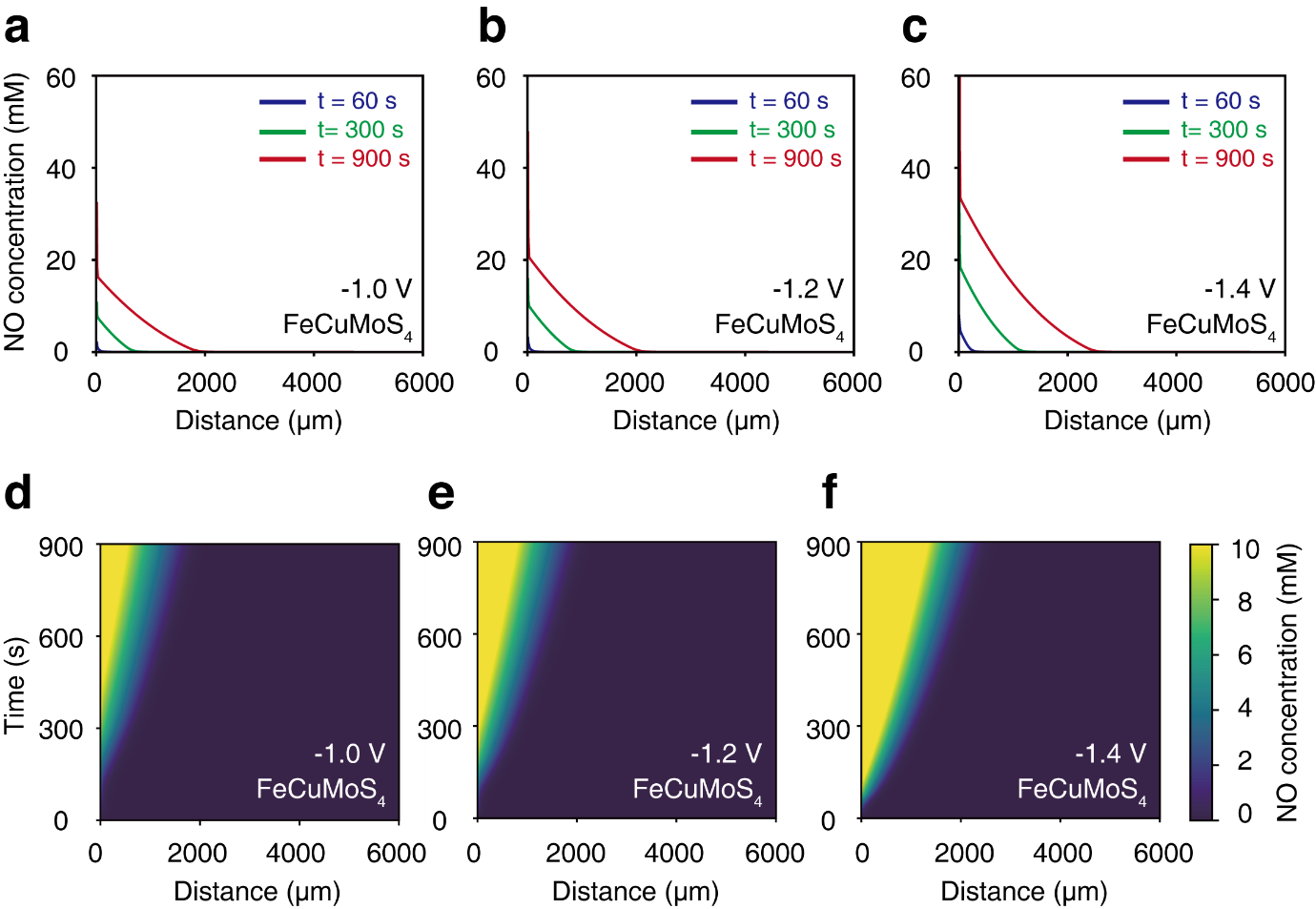


**Figure S13.** Effects of applied voltages on the local concentration profile of NO electrosynthesized with FeCuMoS₄ catalysts. a–c) NO concentration profiles versus distance from the cathode at applied potentials of -1.0 V a), -1.2 V b), and -1.4 V c) versus Ag/AgCl. d–f) Corresponding color maps showing time- and distance-dependent NO distribution at each applied voltage.


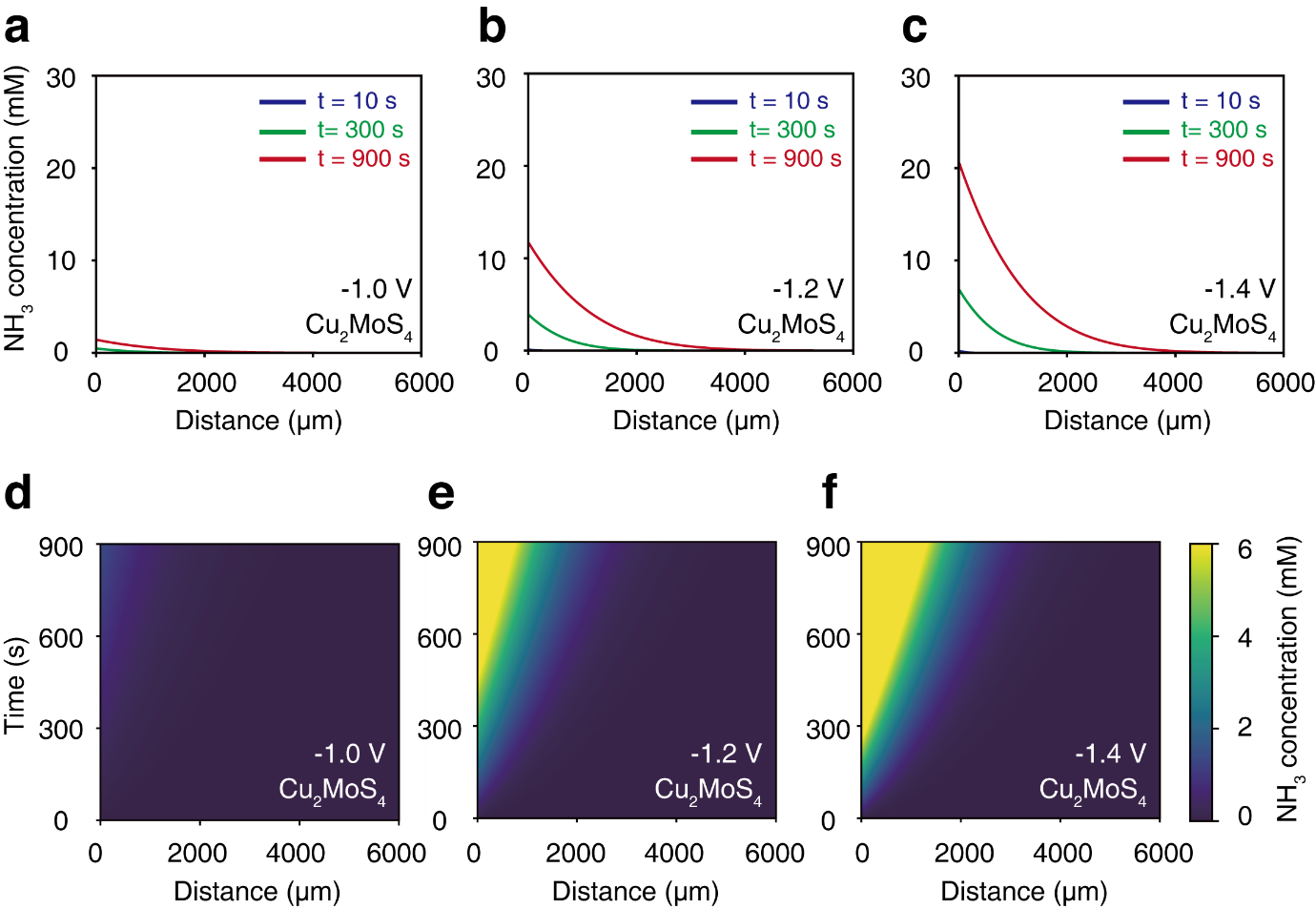


**Figure S14.** Effects of applied voltages on the local concentration profile of NH_3_ electrochemically produced with Cu_2_MoS₄ catalysts. a–c) NH_3_ concentration profiles as a function of distance from the cathode at applied potentials of -1.0 V a), -1.2 V b), and -1.4 V c) versus Ag/AgCl. d–f) Corresponding color maps illustrating the spatiotemporal distribution of NH_3_ at each applied voltage.


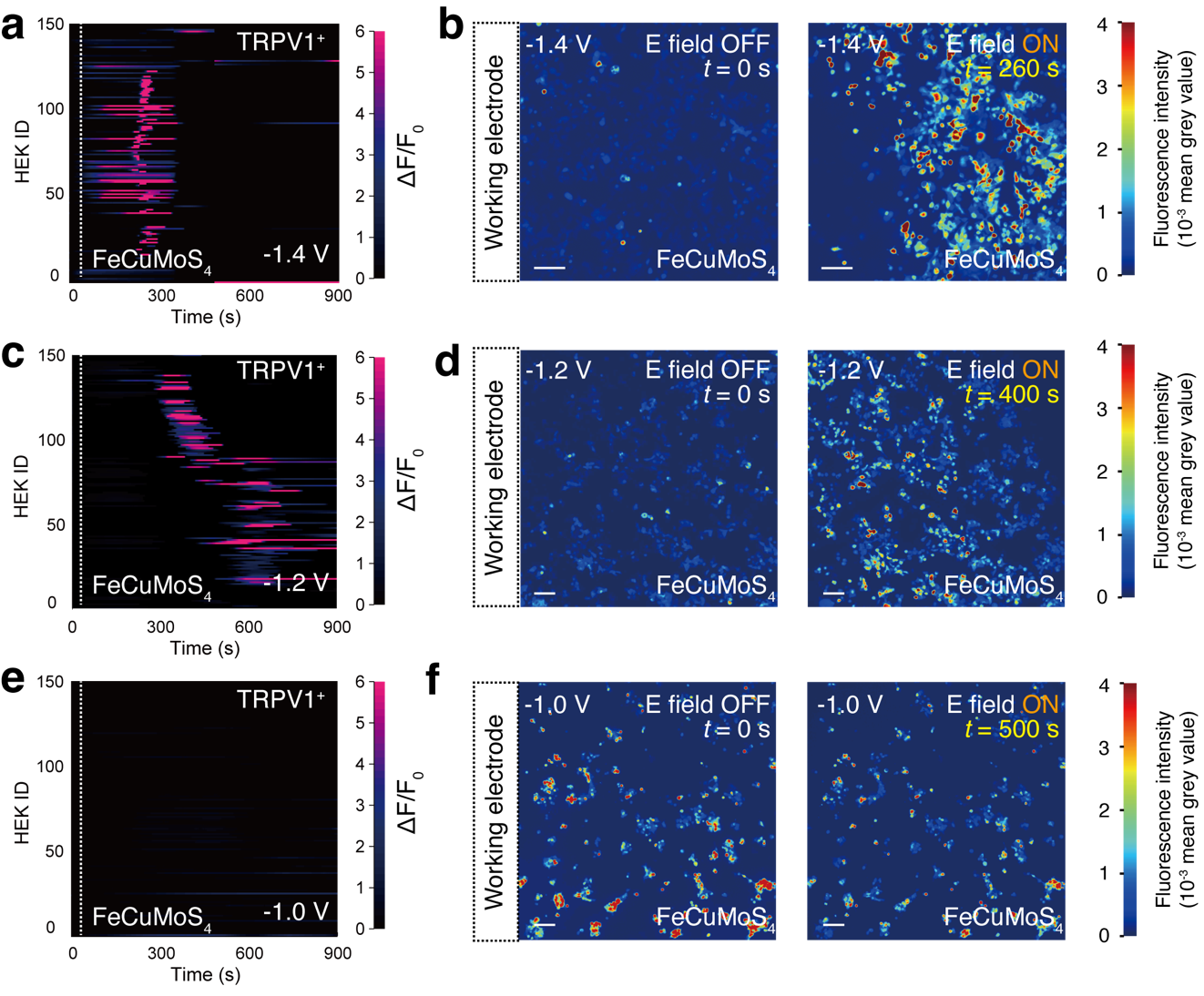


**Figure S15.** Voltage-dependent activation of TRPV1^+^ cells. Individual GCaMP6s fluorescence traces for 150 TRPV1^+^ cells and representative time-lapse images of TRPV1^+^ cells upon application of a-b) -1.4 V, c-d) -1.2 V, and e-f) -1.0 V versus Ag/AgCl, respectively, to FeCuMoS_4_ in the presence of NO_2_^-^ ions (scale bar: 100 μm). Voltages were turned on 30 s (dashed lines) and maintained continuously for electrochemical synthesis until 900 s. More negative voltages facilitated the TRPV1 activation.


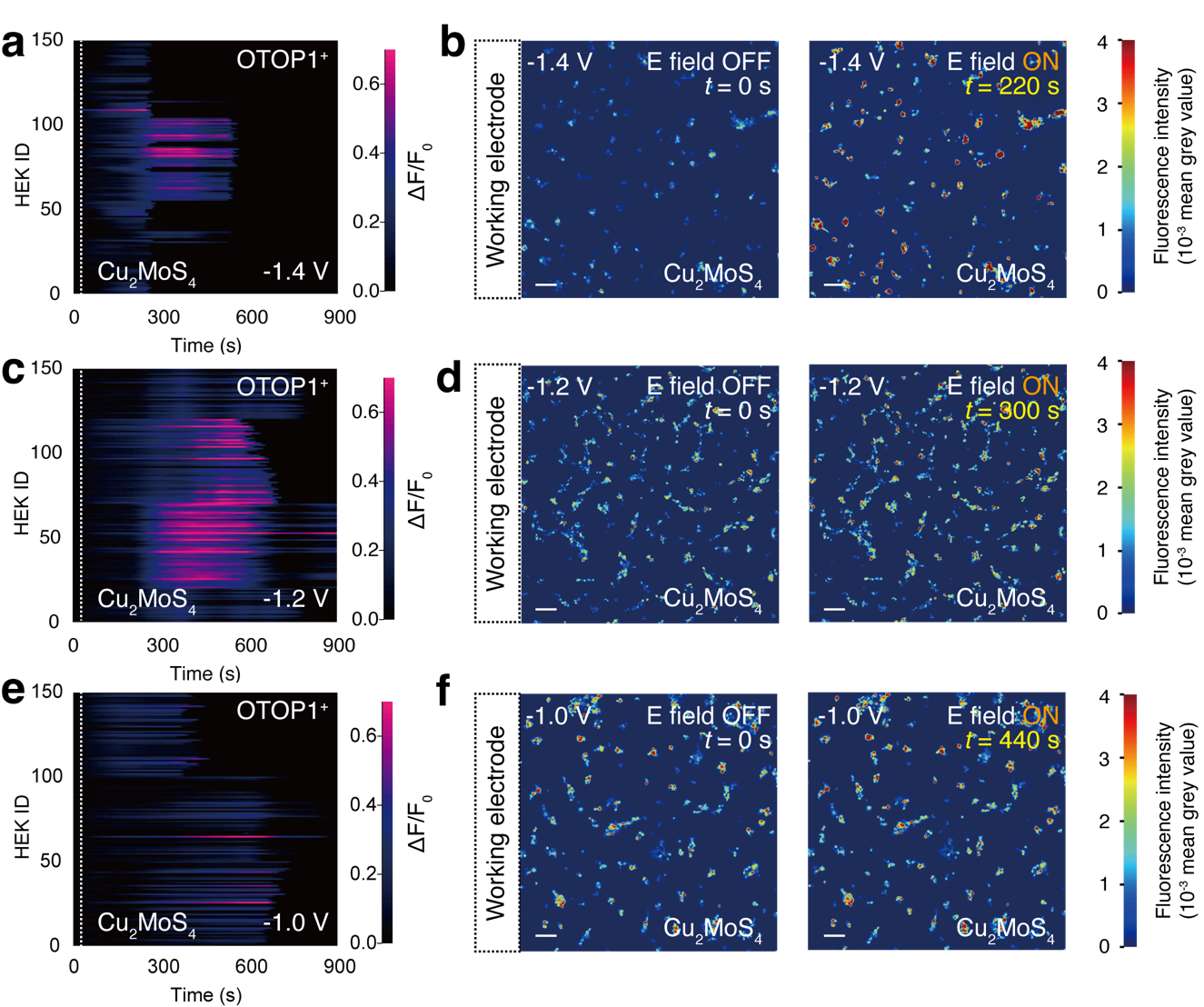


**Figure S16.** Voltage-dependent activation of OTOP1^+^ cells. Individual SEpHluorin fluorescence traces from 150 OTOP1^+^ cells and representative time-lapse images of OTOP1^+^ cells following application of a-b) -1.4 V, c-d) -1.2 V, and e-f) -1.0 V versus Ag/AgCl to Cu_2_MoS_4_ in the presence of NO_2_^-^ ions (scale bar: 100 μm). Voltages were turned on 30 s (dashed lines) and maintained continuously until 900 s. More negative voltages accelerated the activation of OTOP1^+^ cells.


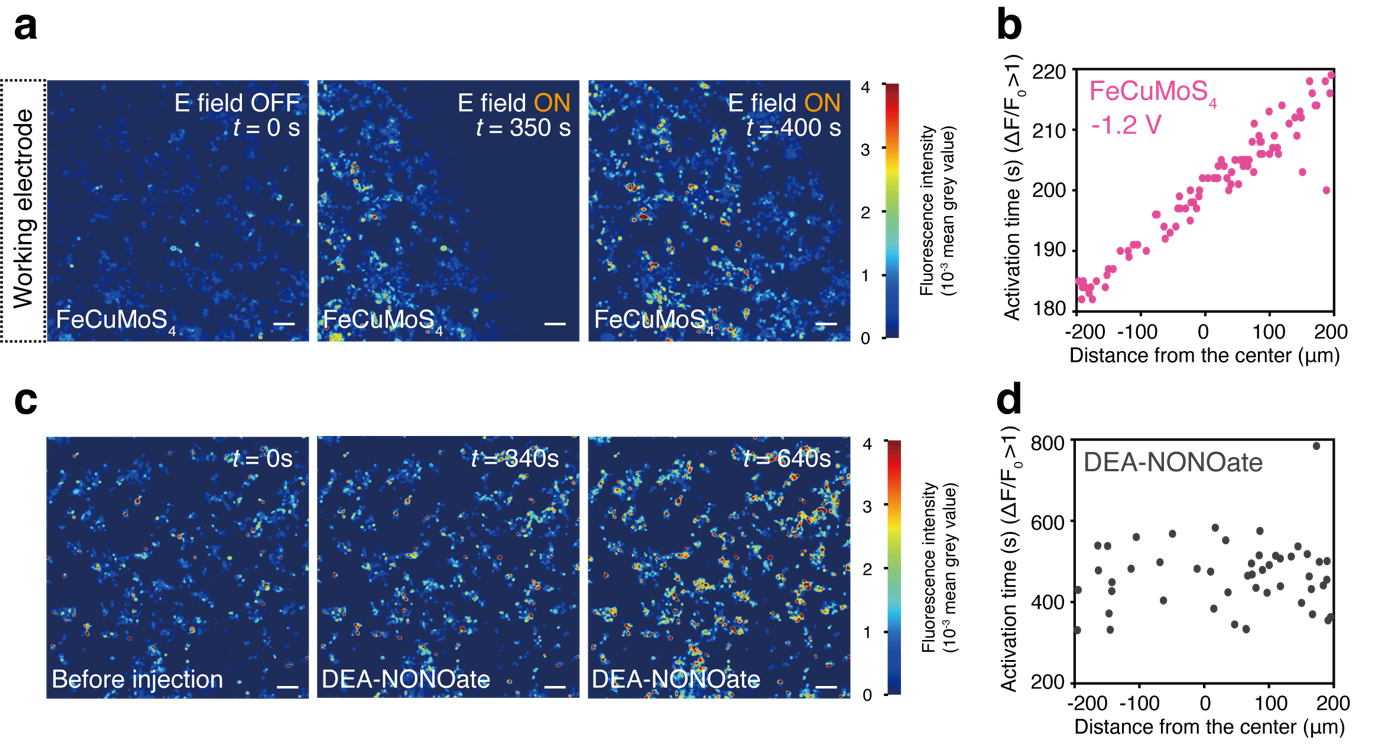


**Figure S17.** Electrolysis time-dependent activation of TRPV1^+^ cells. a) Representative time-lapse images of TRPV1^+^ cells upon application of -1.2 V versus Ag/AgCl to FeCuMoS_4_ electrocatalysts (scale bar: 100 μm), and b) electrolysis time required to activate TRPV1^+^ cells as a function of their distance from the center of the imaging field. Here, negative values on the x-axis indicate TRPV1^+^ cells located closer to the working electrode. c) Representative time-lapse images of TRPV1^+^ cells following the injection of 10 mM DEA-NONOate (scale bar: 100 μm), and d) activation timing of TRPV1^+^ cells as a function of their distance from the center of the imaging field.


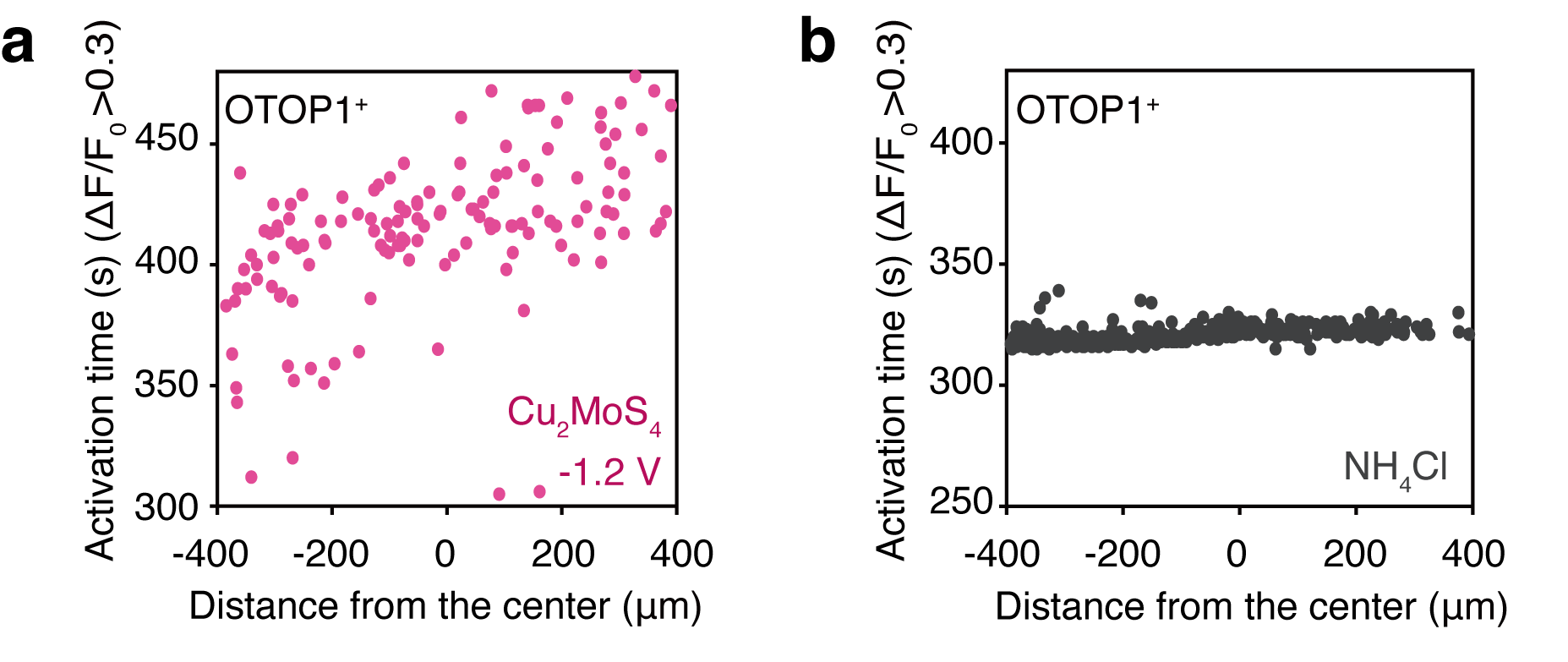


**Figure S18.** Electrolysis time-dependent activation of OTOP1^+^ cells. a) Electrolysis time required to activate OTOP1^+^ cells upon application of -1.2 V versus Ag/AgCl to Cu_2_MoS_4_, plotted as a function of their distance from the center of the imaging field. Here, negative x-axis values indicate OTOP1^+^ cells positioned closer to the working electrode. b) Activation timing of OTOP1^+^ cells following the injection of 10 mM NH_4_Cl as a function of their distance from the center of the imaging field.

**
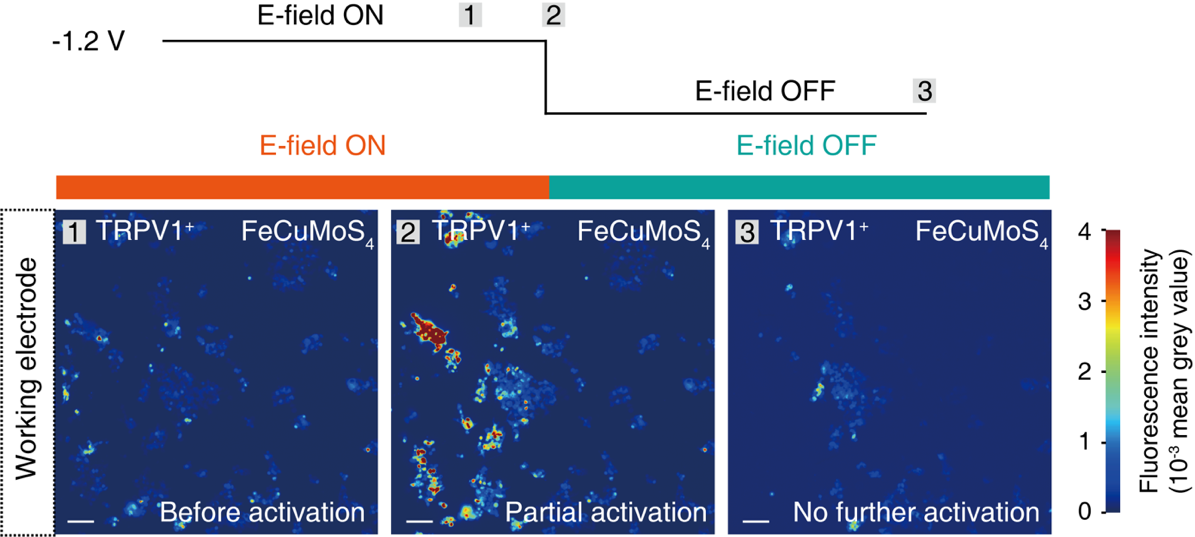
**

**Figure S19.** Effects of applied voltage waveform on biological responses. A step-function-like voltage profile was employed by applying -1.2 V to the FeCuMoS₄ electrocatalysts, followed by abruptly turning off the voltage at point 2. After the voltage was switched off, no further NO-mediated activation of TRPV1⁺ cells was detected at more distant regions (point 3), demonstrating the feasibility of our system for on-demand termination of cellular signaling (scale bar: 100 μm).

**Supplementary Tables**

**Table S1.** Energy calculations for three structures of Fe-substituted Cu_2_MoS_4_ models (Figure S5).

| **Model** | **Energy (eV)** |
| --- | --- |
| a | -264.188 |
| b | -264.185 |
| c | -263.872 |

**Table S2.** The binding energy of NO_2_^-^ at the edge and basal sites of Cu_2_MoS_4_ and FeCuMoS_4_.

| **Electrocatalyst** | **Site** | **Binding energy (eV)** |
| --- | --- | --- |
| Cu_2_MoS_4_ | Edge | -0.57 |
|  | Basal | -0.19 |
| FeCuMoS_4_ | Edge | -0.94 |
|  | Basal | -0.63 |

**Table S3.** Product selectivity of Cu_2_MoS_4_-based electrocatalysts at various voltages.

|  | Cu_2_MoS_4_ | | FeCuMoS_4_ | |  |
| --- | --- | --- | --- | --- | --- |
| ^a^Potential (V) | $\frac{\mathrm{FE}_{NH_{3}}}{\mathrm{FE}_{\mathrm{NO}}}$ | $\frac{\mathrm{FE}_{\mathrm{NO}}}{\mathrm{FE}_{NH_{3}}}$ | $\frac{\mathrm{FE}_{NH_{3}}}{\mathrm{FE}_{\mathrm{NO}}}$ | $\frac{\mathrm{FE}_{\mathrm{NO}}}{\mathrm{FE}_{NH_{3}}}$ | ^b^Ratio |
| -1.0 | 5.71 | 0.18 | 0.19 | 5.29 | 30.22 |
| -1.2 | 33.43 | 0.03 | 0.27 | 3.75 | **125.22** |
| -1.4 | 26.37 | 0.04 | 0.35 | 2.88 | 75.94 |
| -1.6 | 8.07 | 0.12 | 0.37 | 2.69 | 21.72 |

**^a^** Applied potentials (≤ -1.8 V) were excluded from the analysis due to the predominant hydrogen evolution reaction over the NO_2_^-^ reduction reaction.

^b^ Ratio = $\frac{\left( {\mathrm{FE}_{NH_{3}}}/{\mathrm{FE}_{\mathrm{NO}}} \right)_{\mathrm{Cu}_{2}\mathrm{Mo}S_{4}}}{\left( {\mathrm{FE}_{NH_{3}}}/{\mathrm{FE}_{\mathrm{NO}}} \right)_{\mathrm{FeCuMo}S_{4}}}$
